# Selective Up-Regulation of Tumor Suppressor Gene Retinoblastoma by Bisacridine Derivative Through Gene Promoter Quadruplex Structures for Cancer Treatment

**DOI:** 10.3390/ijms26041417

**Published:** 2025-02-07

**Authors:** Xiaomin Lin, Jiahui Zhang, Jihai Liang, Dongsheng Ji, Zhi-Shu Huang, Ding Li

**Affiliations:** School of Pharmaceutical Sciences, Sun Yat-sen University, Guangzhou University City, Guangzhou 510006, China; linxm35@mail2.sysu.edu.cn (X.L.); zhangjh265@mail2.sysu.edu.cn (J.Z.); liangjh225@mail2.sysu.edu.cn (J.L.); jidsh@mail2.sysu.edu.cn (D.J.); ceshzs@mail.sysu.edu.cn (Z.-S.H.)

**Keywords:** retinoblastoma, bisacridine derivative, i-motif, G-quadruplex, transcriptional regulation, cancer

## Abstract

The *retinoblastoma* (RB) gene is an important tumor suppressor gene with a higher mutation frequency than other tumor suppressor genes. The mutation or inactivation of RB has been found in various cancers. The discovery of small molecules to promote RB expression is an effective anti-cancer strategy. Special DNA secondary structures with G-quadruplex and i-motif on the RB promoter could act as “molecular switches” for gene transcriptional regulation and are potentially important targets for the development of new anti-cancer drugs. After extensive screening, we found that the bisacridine derivative **A06** had selective binding and destabilization for both the G-quadruplex and i-motif on the RB promoter, which significantly up-regulated RB gene transcription and translation, resulting in the inhibition of tumor cell proliferation and metastasis. **A06** exhibited potent anti-tumor activity on Hela cells and strongly suppressed tumor growth on the Hela xenograft mice model without significant toxicity. In comparison, **A02** exhibited strong binding and destabilization to the RB promoter G-quadruplex only, which showed a much weaker effect than **A06** on regulating RB expression and producing anti-tumor activity. As we know, this is the first study for up-regulating a tumor suppressor gene through destabilization of both the G-quadruplex and i-motif on the gene promoter, which provides a new strategy for innovative anti-cancer drug discovery and development.

## 1. Introduction

Cancer seriously endangers human life and health and is the leading cause of death for human beings. In 2020, the number of cancer deaths reached three million in China and ten million worldwide [1,2]. The characteristics of tumors can be summarized as follows: sustained proliferative signal, enabling replicative immortality, inducing angiogenesis, avoiding immune destruction, evading growth suppressors, tumor-promoting inflammation, genome instability and mutation, activating invasion and metastasis, deregulating cellular energetics and resisting cell death [3]. Tumor suppressor genes play important roles in suppressing multiple processes of tumor development, especially tumor growth. When the tumor suppressor gene is mutated, its protein can be inactivated, which might lead to the development of a tumor. The *retinoblastoma* (RB) gene is an important tumor suppressor gene, which has a higher mutation frequency than other tumor suppressor genes, with important members including RBL1 and RBL2 [4]. The mutation or inactivation of RB has been found in various cancers such as cervical cancer, osteosarcoma, multiple myeloma, breast cancer, prostate cancer, NSCLC, retinoblastoma, and bladder cancer [5]. The RB protein contains multiple phosphorylation sites, and its phosphorylation will normally result in its functional change [6,7,8]. RB consists of three domains, including the *N*-terminal and *C*-terminal domains, as well as the middle two-pocket domain [9]. RB influences other protein expression mechanisms, usually through the RB-E2F pathway. RB regulates a variety of critical tumor processes, including cell cycle, genomic stability, apoptosis, metabolism, differentiation, angiogenesis, immune response, and cell senescence [10].

**Figure 1:**
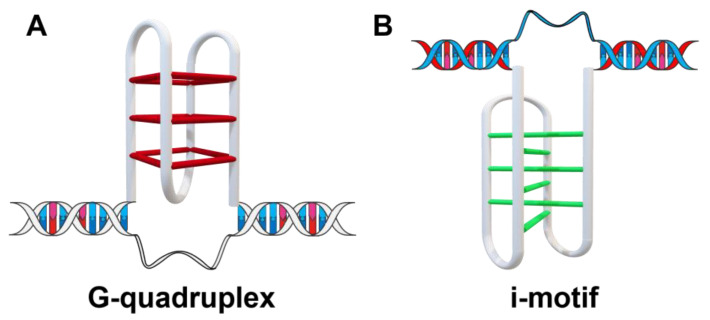
The cytosine and guanine-rich sequences in a single strand of DNA could possibly form quadruplex structures, which could regulate gene replication, transcription, translation, and other important life activities. (**A**) Schematic diagram of the G-quadruplex structure. (**B**) Schematic diagram of C-quadruplex structure named as an intercalated motif or i-motif (IM).

The discovery and development of small molecules for up-regulating RB expression is an effective anti-cancer strategy. Available targeting drugs include mainly CDK inhibitors that inhibit the deactivation of RB phosphorylation, such as Pavosinib [11]; however, it is challenging to restore its protein activity using this method. Firstly, the problem of drug resistance could occur. Secondly, the tumor suppressor gene is usually expressed at a very low level, making it difficult to target via small-molecule drugs. The transcription and translation of the RB gene could be promoted from its original template through gene up-regulation, which could possibly provide an alternative approach without the above protein-level problems. Tumor cells divide much more rapidly in comparison with normal cells, with relatively high-level DNA replication activity. The DNA double helix structure is unfolded to single-strand DNA during replication processes, and other special secondary structures could possibly be formed after unfolding. In particular, cytosine and guanine-rich sequences in single-strand DNA could possibly form quadruplex structures, including the G-quadruplex and i-motif (Figure 1) [12,13]. Quadruplex structures appear mainly in the gene promoter region, the telomere region, and some other important regions and can regulate gene replication, transcription, translation, and other important life activities. G-quadruplex structures from G-rich sequences can be formed under physiological conditions and mainly exist in the S phase of the cell cycle. In comparison, i-motif structures from C-rich sequences are usually formed under molecular crowding conditions or weakly acidic conditions, which mainly exist in the G1 phase [14,15]. The G1 phase is the phase responsible for RNA and ribosome syntheses, which mainly provide the materials and energy required for cells to enter the S phase. Therefore, it can be inferred that G-quadruplex and i-motif have different functional effects on the cell cycle. The quadruplex structures have been regarded as “molecular switches” and could regulate the transcription and translation of genes through their formation and unfolding processes. G-quadruplex has been recognized as a gene suppressor; however, no consensus exists on the role of the i-motif structure. The telomere or promoter G-quadruplex stabilized by small molecules could inhibit telomere elongation or oncogene expression, thus inhibiting tumor growth. On the other hand, it has been shown that the compound IMC-48 can bind to and induce the formation of the Bcl-2 i-motif structure, which results in the up-regulation of Bcl-2 gene expression [16]. In contrast, compound **B19** can stabilize the c-myc gene promoter i-motif structure, resulting in the down-regulation of c-myc transcription and translation [17]. Currently, the Phase I clinical trial of CX-5461 as an anti-cancer drug targeting G-quadruplex has been completed on patients with BRCA1/2 defective tumors [18]; however, a clinical trial for the i-motif binding ligand has not been reported so far.

G/C-rich sequences have been found to exist on the RB gene promoter region, which can form corresponding G-quadruplex and i-motif structures [19,20]. It should be noted that various quadruplex-binding ligands have been studied for down-regulating oncogene transcription and translation against tumor cells. However, it has not been reported to up-regulate tumor suppressor genes by a small-molecule ligand through the gene promoter quadruplex structure. The cells in the body have different cycles, and, therefore, a ligand simultaneously targeting both the G-quadruplex (mainly at the S phase) and i-motif (mainly at the G1 phase) could have greatly increased anti-tumor activity with reduced dosage and side effects. In our present study, after extensive screening, the bisacridine derivative **A06** was found to be a potent dual-binding ligand unfolding both the RB promoter G-quadruplex and i-motif structures. In contrast, the bisacridine derivative **A02** was found to only selectively bind and unfold RB promoter G-quadruplex without strong interactions with its corresponding i-motif. Our molecular level and cellular level experiments showed that **A06** significantly up-regulated RB transcription and translation with potent anti-tumor activity in comparison to **A02**. **A06** also showed excellent anti-tumor activity in human cervical cancer xenografts without significant toxicity and side effects on essential organs. This study also indicates the regulatory roles of RB promoter quadruplex structures and explores their potential as anti-cancer drug targets. As we know, this is the first study to up-regulate a tumor suppressor gene through the destabilization of both the G-quadruplex and i-motif on the gene promoter, which provides a new strategy for innovative anti-cancer drug discovery and development.

## 2. Results

It has been found that G/C-rich sequences exist in the tumor suppressor gene RB promoter region, which can form corresponding G-quadruplex and i-motif structures [19,20]. The RB promoter G-quadruplex has a G-rich sequence of 5′-CGGGGGGTTTTGGGCGGC-3′, while its complementary i-motif has a C-rich sequence of 5′-GCCGCCCAAAACCCCCCG-3′. In the present study, circular dichroism (CD) spectroscopy was used to verify the formation of these G-quadruplex and i-motif structures under our experimental conditions (Figure S1). As shown in Figure S1A,B, the RB promoter i-motif had characteristic i-motif peaks at a pH ranging from 4.4 to 5.7, which confirmed its formation of the i-motif structure. These data show that the RB promoter i-motif was relatively stable under mildly acidic conditions in vitro, which gradually unfolded into a random coil when increasing the pH to near-neutral conditions [21,22,23]. The CD spectrum value at 288 nm for the RB promoter i-motif against pH was used to determine pH_T_ through curve-fitting [24], with pH_T_ determined to be 5.73. pH_T_ is the pH value at which a 50% C-rich sequence forms in the i-motif structure. Based on the above experimental data, the buffer solution at pH 5.5 was chosen for i-motif-relevant experiments to ensure the stability of the i-motif structure. On the other hand, the CD spectrum of the RB promoter G-quadruplex showed a typical parallel G-quadruplex structure in a buffer solution at pH 7.4, as shown in Figure S1C, with a positive absorption peak at 262 nm and a negative absorption peak near 240 nm. This indicated that the RB promoter G-quadruplex could form a relatively stable parallel G-quadruplex structure under normal physiological conditions.

### 2.1. Screening for Small Molecules Binding to RB Promoter Quadruplex Structures

**Figure 2:**
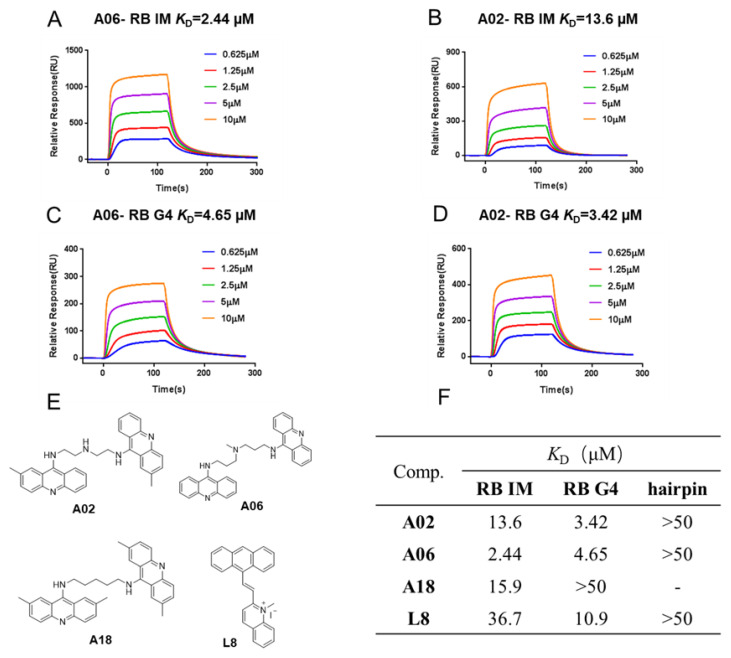
SPR experiments were performed for binding compounds to RB quadruplex structures. (**A**) *K*_D_ value for binding of **A06** to RB promoter i-motif was determined to be 2.44 μM in MES buffer at pH 5.5. (**B**) *K*_D_ value for binding of **A02** to RB promoter i-motif was determined to be 13.6 μM in MES buffer at pH 5.5. (**C**) *K*_D_ value for binding of **A06** to RB promoter G-quadruplex was determined to be 4.65 μM in Tris-HCl buffer at pH 7.4. (**D**) *K*_D_ value for binding of **A02** to RB promoter G-quadruplex was determined to be 3.42 μM in Tris-HCl buffer at pH 7.4. (**E**) Chemical structures of candidate compounds. (**F**) *K*_D_ values for binding of candidate compounds to RB quadruplexes and hairpin structure determined through SPR experiments.

In order to find small-molecule compounds effectively binding to the RB gene promoter i-motif and G-quadruplex structures, the surface plasmon resonance (SPR) experiment was carried out to screen several types of compounds, including acridone derivatives, bisacridine derivatives, quinoline derivatives, benzothiazole derivatives, fluorescent natural product derivatives, etc. Among these compounds, the bisacridine derivative **A06** showed strong binding affinity to the RB promoter i-motif, with its binding constant (*K*_D_) determined to be 2.44 μM. In contrast, the bisacridine derivative **A02** with a similar structure showed weak interaction with the RB promoter i-motif, and the *K*_D_ value was determined to be 13.6 μM (Figure 2A,B). Most other compounds had poor binding affinity to the RB promoter i-motif (*K*_D_ > 50 μM), with the exception of weak interactions for bisacridine derivative **A18** and quinoline derivative **L8** (Figure 2). Then, the binding constants of these compounds to the RB promoter G-quadruplex were determined using the SPR experiment. The binding affinities of these compounds to the hairpin structure were also measured since their interactions could possibly increase the toxicity or side effects of drug molecules through intercalation. As shown in Figure 2, both **A06** and **A02** showed a strong binding affinity to the RB promoter G-quadruplex, with their *K*_D_ values determined to be 4.65 μM and 2.44 μM, respectively, without significant interactions with the hairpin structure. Based on the above SPR data, the bisacridine derivative **A06** had strong binding affinity to both the RB promoter i-motif and G-quadruplex, while the bisacridine derivative **A02** with a similar structure had strong binding affinity to the RB promoter G-quadruplex only.

The MST experiment was also performed to further study and confirm the binding affinity of **A06** and **A02** to RB promoter quadruplex structures. **A06** showed excellent binding affinity to the RB promoter i-motif and G-quadruplex with its *K*_D_ values determined to be 0.43 ± 0.24 μM and 2.50 ± 0.65 μM, respectively, as shown in Figure S2A,B. The *K*_D_ values for the binding of **A02** to RB promoter quadruplex structures were determined to be 18.06 ± 5.28 μM for the RB promoter i-motif and 0.50 ± 0.27 μM for the RB promoter G-quadruplex, as shown in Figure S2C,D, indicating its strong binding to the RB promoter G-quadruplex only. Our MST experimental data were consistent with the above SPR experimental results. Therefore, in the present study, the bisacridine derivative **A06**, with dual interactive effects, was selected for further in-depth study, and the bisacridine derivative **A02** with a similar structure targeting the G-quadruplex only served as a control compound for comparison.

### 2.2. Bisacridine Derivative ***A06*** and ***A02*** Could Destabilize RB Promoter Quadruplex Structures

In order to further understand the interactions of the bisacridine derivatives **A06** and **A02** with RB promoter quadruplex structures, we conducted a CD experiment. As shown in Figure S3A, with the addition of an increasing amount of compound **A06**, the positive peak at 288 nm gradually shifted toward 280 nm with its intensity gradually decreased, while the negative peak at 264 nm disappeared, indicating that **A06** had a good interaction with the RB promoter i-motif [22,25,26]. For the RB promoter G-quadruplex, its characteristic peak at 260 nm decreased upon the addition of **A06**, indicating that **A06** also had an interaction with the RB promoter G-quadruplex (Figure S3B). In comparison, upon the addition of **A02**, the CD spectrum of the RB promoter i-motif had only a little change, with the peak at 288 nm decreasing slightly, indicating weak interactions. For the RB promoter G-quadruplex, upon the addition of **A02**, its CD spectrum changed significantly while its characteristic peak at 260 nm decreased markedly, indicating strong interactions (Figure S3C,D). Our above results show that **A06** interacted with both the RB promoter i-motif and G-quadruplex, while **A02** had good interactions with the RB promoter G-quadruplex only. These CD data are consistent with the binding study results from SPR and MST experiments.

CD melting experiments can be used to evaluate the thermal stability of the quadruplex structure. When the temperature rises, the quadruplex structure expands and forms a single chain [27]. In our CD melting experiment, **A06** showed good disassembling ability when unfolding both the RB promoter i-motif and G-quadruplex with its Δ*T*_m_ values determined to be −10.7 °C for the RB promoter i-motif and −6.4 °C for the RB promoter G-quadruplex (Figure S3E,F). In comparison, **A02** showed good disassembling ability when unfolding the RB promoter G-quadruplex only, with its Δ*T*_m_ value determined to be −9.9 °C for the RB promoter G-quadruplex (Figure S3E,F).

The FRET experiment was also performed to study the effects of compounds **A06** and **A02** on the stability of RB gene promoter quadruplex structures. The oligomers for the FRET experiment were dual-labeled with FAM at the 5′ end and TAMRA at the 3′ end. FAM is 6-carboxyfluorescein as donor fluorophore, and TAMRA is 6-carboxytetramethylrhodamine as acceptor fluorophore. The excitation and emission wavelengths of FAM were 480 nm and 518 nm, respectively, and those of TAMRA were 560 nm and 585 nm, respectively. When the secondary structure was formed, these two fluorescent groups were close to each other, leading to a significantly increased absorption peak at 585 nm for the fluorescent response of TAMRA due to its energy received from FAM. On the contrary, when the secondary structure was disassembled, the fluorescence decreased at 585 nm [28]. Our experimental data show that this method could be adequately applied to the study of the RB promoter i-motif and G-quadruplex (Figure S4A,B). As shown in Figure S4C, with the addition of an increasing amount of compound **A06**, the fluorescent response of TAMRA gradually decreased in a dose-dependent manner, indicating that the fluorescent receptor and donor were separated from each other. Similarly, with the addition of an increasing amount of **A02** to the RB promoter i-motif, the fluorescent response of TAMRA was reduced in a dose-dependent manner, indicating that **A02** could also unfold the RB promoter i-motif structure but with a weaker effect than **A06** (Figure S4D). Both **A06** and **A02** showed disassembling ability when unfolding the RB promoter G-quadruplex structure, and the disassembling ability of **A02** was stronger than that of **A06** (Figure S4E,F). As shown in Figure S4G, our FRET experiment shows that **A06** can unfold both the RB promoter i-motif and G-quadruplex, with a stronger effect on the RB promoter i-motif. Our FRET experiment also indicated that **A02** can unfold both the RB promoter i-motif and G-quadruplex, with a stronger effect on the RB promoter G-quadruplex. FRET is a much more sensitive experiment with fluorescent detection using significantly lower amounts of the labeled DNA oligomer. Our above FRET experimental data are generally consistent with previous experimental results, which further confirms the disassembling ability of **A06** and **A02** for unfolding RB promoter quadruplex structures.

### 2.3. Bisacridine Derivative ***A06*** Could Specifically Bind to and Destabilize RB Promoter Quadruplex Structures

To evaluate whether the bisacridine derivative **A06** could specifically bind to RB promoter quadruplex structures, SPR and TO displacement experiments were carried out to measure its binding affinity to some other gene promoter quadruplex structures for comparison. Our SPR experimental data (Figure S5) showed that the *K*_D_ values for its binding to promoter quadruplex structures of *ACC2*, *ACC1*, *bcl-2*, *RET*, *c-jun*, *braf*, and *kras* were estimated to be in the range of 15–55 μM, indicating its much weak binding interactions with these DNA promoter quadruplex structures. The TO displacement experiment was also performed to evaluate the binding specificity of compounds to quadruplex structures. The TO displacement ratio is proportional to the binding ability of compounds to quadruplex structures. DC_50_ refers to the concentration of the compound at a displacement ratio of 50%. As shown in Figure S6A,B, with the addition of an increasing amount of **A06**, the fluorescence intensity at 530 nm significantly decreased in a dose-dependent manner, indicating that **A06** could competitively bind to the RB promoter i-motif and G-quadruplex. The DC_50_ values of **A06** for its interactions with the RB promoter i-motif and G-quadruplex were determined to be 3.4 μM and 2.7 μM, respectively (Figure S6C). Our TO displacement result showed that the displacement ability of **A06** to the RB quadruplex was significantly stronger than that of others (Figure S6D), indicating that **A06** had good binding selectivity to RB promoter quadruplex structures.

A CD experiment was carried out to study the interaction of **A06** with other gene promoter quadruplex structures. As shown in Figure S7, the addition of **A06** had no significant effect on other gene promoter quadruplex structures, indicating that **A06** could specifically unfold RB promoter quadruplex structures without a clear effect on the quadruplex structures of other gene promoters. The FRET experiment was also performed to further verify its binding specificity. The effects of **A06** on some other gene promoter structures, including the *Hif-1α* i-motif, telomere (Tel) i-motif, *kras* i-motif, *VEGF* G-quadruplex, *bcl-2* i-motif, and *c-myc* i-motif were determined using the FRET experiment. It was found that the addition of **A06** had no significant effect on their fluorescence intensity at 585 nm (Figure S8). In summary, our above results show that **A06** could specifically bind to and unfold RB promoter i-motif and G-quadruplex structures.

### 2.4. Further Studies of Interactions Between Bisacridine Derivative ***A06*** and RB Promoter Quadruplex Structures

An ultraviolet–visible (UV) experiment was performed to further study the interaction between the **A06** and RB promoter quadruplex structures. As shown in Figure S9, with the addition of an increasing amount of the RB promoter i-motif or G-quadruplex, hypochromic effect and red-shift were observed for the UV absorption peak at 410 nm on the spectra. After the addition of 5 μM of the oligomer for the RB promoter i-motif or G-quadruplex, the hypochromic ratio for the UV absorption peak was about 38.7% with a red-shift of 5 nm. The phenomenon of red-shift is usually caused by the conjugation of molecules or the introduction of the auxochrome group. The hypochromic effect and red-shift for the UV absorption peak in the present study could be due to the binding of compound **A06** to the quadruplex structure, leading to the accumulation and further delocalization of π electrons [29,30,31], which might also indicate π-π stacking interactions between compound **A06** and RB quadruplex structures.

The EMSA experiment was also carried out to study the interactions between **A06** and RB promoter quadruplex structures. As shown in Figure S10, the oligomer for the RB promoter i-motif had two migration bands on the native gel in the BPES buffer at pH 5.5, with the slow-running band existing as the major band on top of the gel in the form of the i-motif structure. Upon increasing the concentration of **A06**, the top i-motif band gradually decreased, while the bottom band corresponding to its single-strand oligomer increased in a dose-dependent manner, which indicates that **A06** can unfold the RB promoter i-motif structure to give its single-strand oligomer. Similarly, the oligomer for the RB promoter G-quadruplex had two migration bands on the native gel in Tris-HCl buffer at pH 7.4, with the slow-running band existing as the major band on top of the gel in the form of the G-quadruplex structure. Upon the addition of an increasing concentration of **A06**, the top G-quadruplex band gradually decreased, while the bottom band corresponding to its single-strand oligomer increased in a dose-dependent manner, which indicated that **A06** could also unfold the RB promoter G-quadruplex structure to give its single-strand oligomer. It appeared that **A06** had a much stronger unfolding effect on the RB promoter i-motif rather than G-quadruplex.

**Figure 3:**
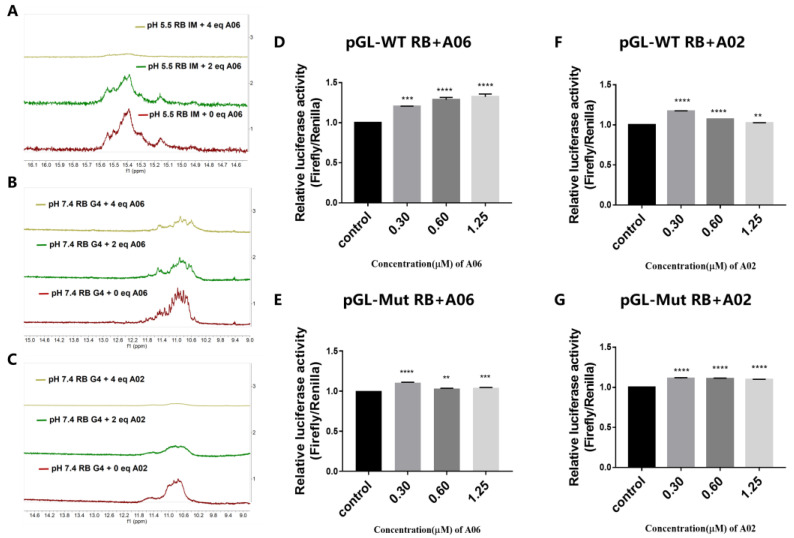
NMR was performed to bind **A06** or **A02** to RB promoter quadruplexes with, and their effects on gene transcriptions were studied using dual luciferase reporter assay. (**A**) NMR spectra of RB promoter i-motif structure with addition of different equivalents of **A06** in BPES buffer at pH 5.5. (**B**) NMR spectra of RB promoter G-quadruplex structure with addition of different equivalents of **A06** in Tris-HCl buffer at pH 7.4. (**C**) NMR spectra of RB promoter G-quadruplex structure with addition of different equivalents of **A02** in Tris-HCl buffer at pH 7.4. (**D**) Effect of **A06** on luciferase expression of pGL-WT RB plasmid. (**E**) Effect of **A06** on luciferase expression of pGL-Mut RB plasmid. (**F**) Effect of **A02** on luciferase expression of pGL-WT RB plasmid. (**G**) Effect of **A02** on luciferase expression of pGL-Mut RB plasmid. Experiments were repeated three times, with data shown as mean ± SEM. ** means *p* < 0.01, *** means *p* < 0.001, and **** means *p* < 0.0001.

The NMR titration experiment was also performed to verify the interactions between **A06** and RB promoter quadruplex structures. The i-motif structure contained multiple semi-protonated C-C^+^ base pairs, which were formed through hydrogen bonding. In ^1^H NMR experiments, the imino protons in C-C^+^ base pairs of the i-motif structure have characteristic peaks at 15–16 ppm [32,33]. The characteristic peaks of the G-quadruplex were around 10.5–12 ppm [34]. The peak height of the characteristic peak can reflect the amount of structure formation in the solution. As shown in Figure 3A, the imino proton peaks at around 15−16 ppm for the RB promoter i-motif were significantly decreased upon the addition of compound **A06** in the BPES buffer at pH 5.5, indicating that **A06** could effectively unfold the RB promoter i-motif structure. Similarly, the characteristic peaks of the RB promoter G-quadruplex around 10.5–12 ppm were decreased upon the addition of compounds **A06** and **A02** in the Tris-HCl buffer at pH 7.4 (Figure 3B,C), indicating that both **A06** and **A02** could unfold the RB promoter G-quadruplex structure. Our experimental data showed that **A02** had a stronger unfolding effect on the RB promoter G-quadruplex than **A06**.

The ESI-MS experiment was also carried out to confirm the interactions between **A06** and RB promoter quadruplex structures. As shown in Figure S11A, the oligomer for the RB promoter i-motif showed a mass signal peak at 5359 Da in the BPES buffer at pH 5.5. Upon the addition of 2 eq **A06**, the mass signal peak at 5849.5 Da for the RB promoter i-motif with **A06** appeared, as shown in Figure S11B, indicating that **A06** could tightly bind to the RB promoter i-motif under acidic conditions. The molecular weight of **A06** was 499.66, and some hydrogen atoms could be lost on **A06** or the DNA phosphate group during their interaction. In comparison, the mass signal peak for the RB promoter i-motif with **A06** did not appear upon the addition of **A06** to the RB promoter i-motif in the BPES buffer at pH 6.6, as shown in Figure S11C,D. This indicates that **A06** could not bind tightly with single-strand oligomers under near-neutral conditions. On the other hand, the oligomer for the RB promoter G-quadruplex showed a mass signal peak at 5642 Da in the Tris-HCl buffer at pH 7.4 (Figure S11E). Upon the addition of 2 eq **A06**, the mass signal peak at 6126.3 Da for the RB promoter G-quadruplex with **A06** appeared, as shown in Figure S11F, indicating that **A06** could tightly bind to the RB promoter G-quadruplex under near-neutral conditions. The theoretical mass signal peak for the RB promoter G-quadruplex with **A06** should be 6143 Da and a hydroxyl group of 17 Da could be lost during the interaction. In summary, our above results further confirm the interactions of **A06** with the RB promoter i-motif and G-quadruplex structures.

### 2.5. ***A06*** Up-Regulated RB Gene Transcription and Translation in Hela Cells

Since **A06** could bind to the RB promoter quadruplex, we investigated whether these binding interactions could consequently affect RB gene transcription and translation. To study the effect of **A06** on RB promoter activity, a dual luciferase reporter experiment was carried out. Luciferase with the RB promoter wild type and a deleted and mutant sequence were constructed. Firstly, the RB promoter region, including its i-motif/G-quadruplex forming sequence, was selected and named wild-type RB (WT RB). On the basis of the WT RB sequence, RB with deletion (Del RB) was designed by deleting the i-motif/G-quadruplex forming sequence without changing other sequences. Similarly, based on the WT RB sequence, its i-motif/G-quadruplex forming sequence was mutated and named Mutant RB (Mut RB). The wild-type RB sequence (WT RB), RB sequence with deletion (Del RB), or Mutant RB sequence (Mut RB) was inserted into luciferase pGL-3 basic plasmid to construct pGL-WT RB, pGL-Del RB, or pGL-Mut RB plasmid, respectively, which was then transfected into Hela cells. After incubation for 24 h, increasing concentrations of **A06** or **A02** were added, followed by further incubation for 48 h. As shown in Figure 3D, for pGL-WT RB containing both the RB promoter i-motif and G-quadruplex, the addition of compound **A06** up-regulated the relative firefly luciferase expression level in a dose-dependent manner. For pGL-Del RB with the deletion of the RB promoter i-motif/G-quadruplex forming sequence, the addition of compound **A06** slightly reduced the relative firefly luciferase expression level (Figure S12A). For pGL-Mut RB with the mutation of the RB promoter i-motif/G-quadruplex-forming sequence, compound **A06** had no significant effect on the relative firefly luciferase expression level (Figure 3E). These data indicate that **A06** could regulate RB promoter activity through its interaction with promoter i-motif and G-quadruplex structures. In contrast, the relative firefly luciferase expression levels for the above plasmids showed no significant change upon the addition of compound **A02** (Figure 3). Our previous results demonstrated that **A06** could unfold both RB promoter i-motif and G-quadruplex structures with a stronger effect on the RB promoter i-motif, while **A02** could unfold the RB promoter G-quadruplex only without a significant effect on the RB promoter i-motif. Our dual luciferase reporter experiment showed that **A06** was significantly better than **A02** at regulating RB promoter activity by simultaneously unfolding both i-motif and G-quadruplex structures on the RB promoter region.

**Figure 4:**
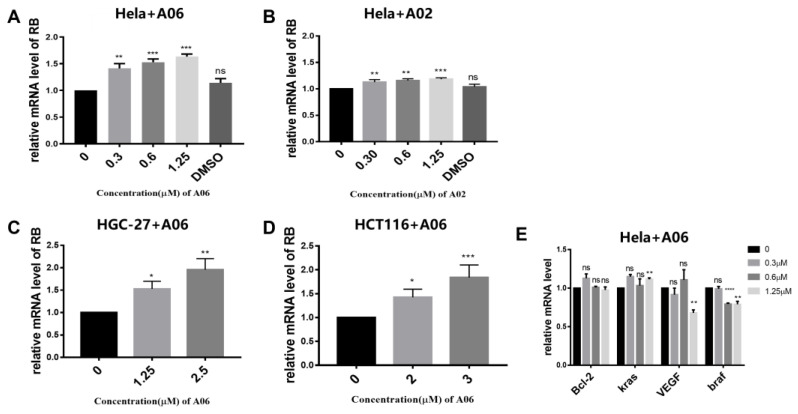
The qPCR experiment was performed to analyze the effects of **A06** and **A02** on RB gene transcription in cells. (**A**) The transcription levels of RB mRNA were measured for Hela cells incubated with increasing concentrations of **A06** for 24 h. (**B**) The transcription levels of RB mRNA were measured for Hela cells incubated with increasing concentrations of **A02** for 24 h. (**C**) The transcription levels of RB mRNA were measured for HGC-27 cells incubated with increasing concentrations of **A06** for 24 h. (**D**) The transcription levels of RB mRNA were measured for HCT116 cells incubated with increasing concentrations of **A06** for 24 h. (**E**) The mRNA levels of other genes were measured for Hela cells incubated with compound **A06** at increasing concentrations for 24 h. The experiments were repeated three times, with data shown as mean ± SEM. ns means non-significant, * means *p* < 0.05, ** means *p* < 0.01, *** means *p* < 0.001, and **** means *p* < 0.0001.

In order to further analyze the effect of **A06** on gene transcriptions, we conducted quantitative real-time PCR experiments on Hela, HGC-27, and HCT116 cells treated with **A06** for 24 h. As shown in Figure 4, RB mRNA levels were found to be significantly increased in dose-dependent manners on three types of cells, possibly through the binding and unfolding of RB promoter quadruplex structures. In contrast, **A02** had a very weak effect on regulating RB transcription in Hela cells (Figure 4B). This further confirmed that **A06** dual-targeting both the RB promoter i-motif and G-quadruplex could regulate RB transcription much more effectively than **A02** targeting the RB promoter G-quadruplex only. The effects of **A06** on other gene transcriptions were also studied, as shown in Figure 4E, which indicated that **A06** had no significant regulatory effect on these gene transcriptions. These data suggest that **A06** specifically regulated RB transcription, possibly through its selective interactions with RB promoter i-motif and G-quadruplex structures.

**Figure 5:**
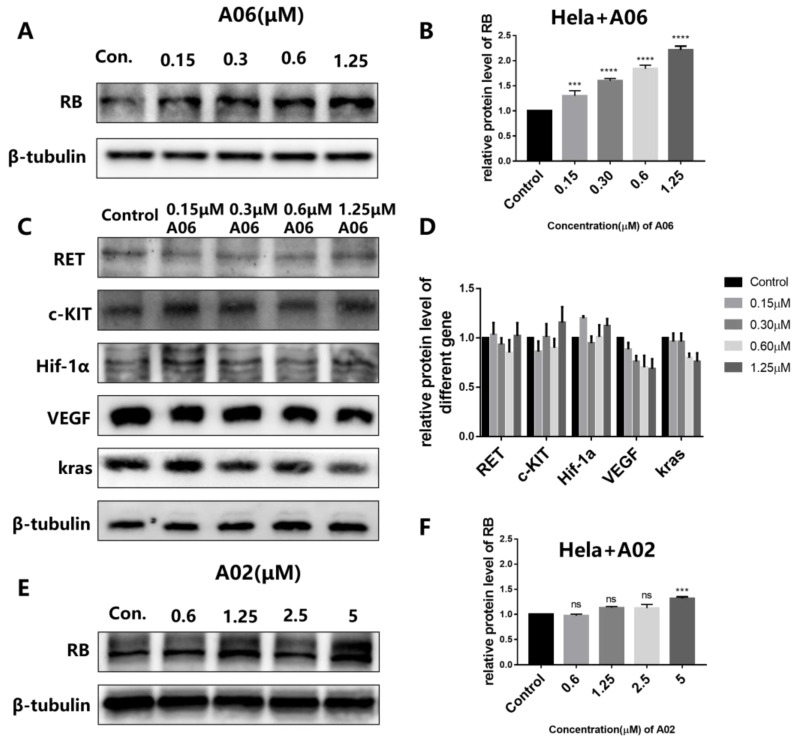
The protein expressions in Hela cells were analyzed using Western blot. (**A**) The expression levels of the RB protein in Hela cells incubated with increasing concentrations of **A06** for 24 h. (**B**) A histogram of the gray values of WB strips is in (**A**) for analysis. (**C**) The expression levels of other proteins in Hela cells incubated with increasing concentrations of **A06** for 24 h. (**D**) A histogram of the gray values of WB strips is in (**C**) for analysis. (**E**) The expression levels of the RB protein in Hela cells incubated with increasing concentrations of **A02** for 24 h. (**F**) A histogram of gray values of WB strips is in (**E**) for analysis. Experiments were repeated three times, with data shown as mean ± SEM. ns means non-significant, *** means *p* < 0.001, and **** means *p* < 0.0001.

The effects of **A06** on expressions of RB and some other related proteins were determined using Western blot. As shown in Figure 5A,B, **A06** could significantly increase RB protein expression in a dose-dependent manner in Hela cells without significant effect on the expressions of other analyzed proteins, including RET, c-Kit, Hif-1α, VEGF, and kras, as shown in Figure 5C,D. In comparison, **A02** had no significant effect on RB expression, as shown in Figure 5E,F. These results further confirm the fact that **A06** interacting with both the RB promoter i-motif and G-quadruplex structures had a much more significant effect on the regulation of RB expression. The regulatory effects of **A06** on RB expression in other types of cancer cells were also studied. The addition of **A06** significantly up-regulated the expression of the RB protein in HGC-27 (Figure S12C,D) and U2OS cells (Figure S12E,F). The effect of **A06** on RB protein expression was also determined using immunofluorescence. Immunofluorescence technology can be used to detect and locate target antigens by binding to specific antibodies labeled with a fluorophore for signal amplification with high sensitivity [35]. As shown in Figure S13, green fluorescence with the indication of RB protein expression in Hela cells gradually increased upon the addition of the increasing concentration of **A06**. These results indicate that **A06** could selectively up-regulate RB gene expression in tumor cells, possibly through the binding and unfolding of the RB promoter i-motif and G-quadruplex structures.

### 2.6. ***A06*** Induced Cancer Cell Apoptosis and Inhibited Cell Proliferation and Metastasis

**Figure 6:**
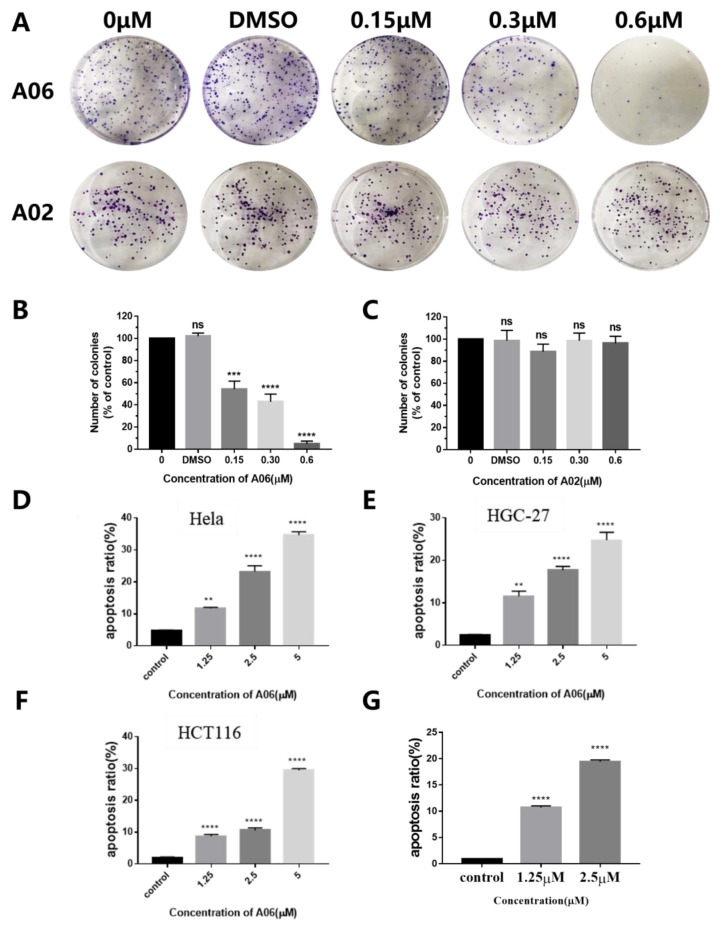
Compound **A06** inhibited cancer cell proliferation and induced cell apoptosis. (**A**) The colony formation assay was performed for Hela cells incubated with increasing concentrations of compounds **A06** or **A02** for 9 days. (**B**) The histogram was plotted for the number of Hela cell colonies after incubation with increasing concentrations of compound **A06** for 9 days. (**C**) The histogram was plotted for the number of Hela cell colonies after incubation with increasing concentrations of compound **A02** for 9 days. (**D**) The apoptosis ratio of Hela cells was plotted after incubating with compound **A06** at increasing concentrations for 48 h. (**E**) The apoptosis ratio of HGC-27 cells was plotted after incubating with compound **A06** at increasing concentrations for 48 h. (**F**) The apoptosis ratio of HCT116 cells was plotted after incubating with compound **A06** at increasing concentrations for 48 h. (**G**) The histogram for the Hoechst staining of Hela cells treated with **A06**, with cell apoptosis gradually increasing in a dose-dependent manner. Experiments were repeated three times, with data shown as mean ± SEM. ns means non-significant, ** means *p* < 0.01, *** means *p* < 0.001, and **** means *p* < 0.0001.

The MTT assay was performed to evaluate the activity of **A06** against human cancer cells, which also provided a certain data basis and concentration reference for later cellular and animal experiments. As shown in Table S5, compound **A06** had strong inhibition on the proliferation of eight types of tumor cells (IC_50_ < 3 μM), indicating that **A06** could become a broad-spectrum anti-tumor lead compound. In comparison, the anti-tumor activity of **A02** was much weaker than that of **A06**. To further evaluate the effect of **A06** on the growth inhibition of cancer cells, a colony formation assay was performed. As shown in Figure 6A,B, the number of cell clones was reduced significantly in a dose-dependent manner upon treatment with **A06**. In contrast, **A02** had no significant effect on the number of cell clones (Figure 6A,C). Our above results show that the ability of **A06** to inhibit the growth of cancer cells was significantly stronger than that of **A02**, which is possibly due to its ability to unfold both the structures of the RB promoter i-motif and G-quadruplex.

To quantitatively analyze the effect of compound **A06** on the apoptosis of various types of tumor cells, the Annexin V-FITC/PI double staining experiment was carried out. Our apoptosis experiment (Figure S14A–C) showed that **A06** causes a significant increase in cell apoptosis on Hela cells in a dose-dependent manner from 4.62% without **A06** treatment to 34.76% with 5 μM **A06** treatment (Figure 6D). **A06** also causes significant increases in cell apoptosis in a dose-dependent manner on HGC-27 cells and HCT116 cells (Figure 6E,F). These results demonstrate that **A06** can induce the apoptosis of various types of tumor cells. Hoechst staining is another method to detect apoptosis, which is simple and rapid. Hoechst 33342 can penetrate cell membranes and bind to nuclear DNA with low toxicity to cells. After Hoechst staining, the nuclei of normal cells were turned blue under a fluorescence microscope. Due to chromatin shrinkage, the nuclei of apoptotic cells were dense and heavily stained. As shown in Figure S14D, Hoechst staining images were taken for Hela cells incubated with compound **A06** at increasing concentrations for 48 h, with the nuclei of the cells turned blue. For the nuclei of the cells treated with **A06**, some bright blue cells, due to chromatin shrinkage, gradually appeared in a dose-dependent manner with a histogram, as shown in Figure 6G, which further demonstrates that **A06** could induce apoptosis of Hela cells.

**Figure 7:**
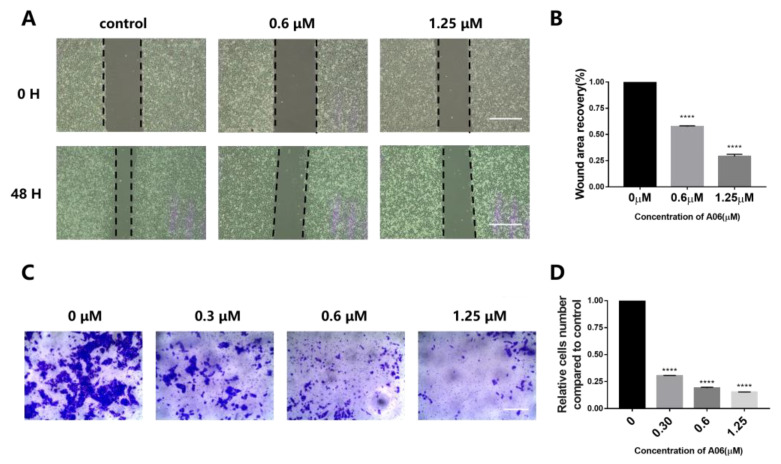
Compound **A06** inhibited the migration and invasion of Hela cells. (**A**) A wound healing experiment was performed, which showed that **A06** significantly inhibited the migration of Hela cells (Scale bars: 200 μm). (**B**) **A06** significantly inhibited the migration of Hela cells, with the comparison of wound area recovery values. (**C**) A transwell assay experiment was carried out with representative pictures (Scale bar: 200 μm). (**D**) The transwell assay experiment showed that the invasion number of Hela cells in the **A06** treatment groups significantly decreased. Experiments were repeated three times, with data shown as mean ± SEM. **** means *p* < 0.0001.

In order to study the effect of **A06** on the migration or metastasis of Hela cells, a wound-healing experiment was performed, as shown in Figure 7A,B. Our experiment showed that the scratch area of the cells decreased significantly after 48 h without **A06** treatment, with cell migration clearly occurring. With the addition of an increasing amount of **A06**, the migration of Hela cells was significantly inhibited in a dose-dependent manner. A transwell assay experiment was also carried out, and the results are shown in Figure 7C,D. Compared with the control group, the invasion number of Hela cells in **A06** treatment groups significantly decreased in a dose-dependent manner. These experiments show that **A06** has a significant effect on the migration or metastasis of Hela cells.

### 2.7. ***A06*** Inhibited Tumor Growth in a Human Cervical Cancer Xenograft

**Figure 8:**
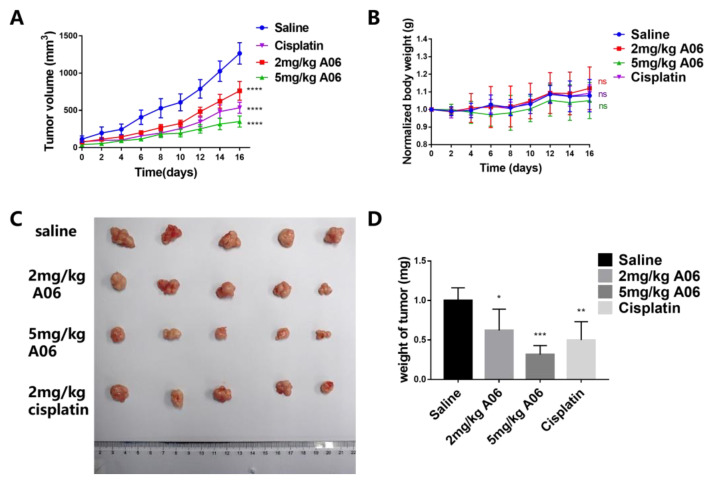
The anti-tumor effect of compound **A06**. (**A**) Tumor volumes (mm^3^) were recorded for the mice in the 5 mg/kg **A06** group, 2 mg/kg **A06** group, saline group, and cisplatin group. (**B**) Changes in the body weight of mice during administration. (**C**) Tumor entity images. (**D**) The tumor weights of mice in the above four groups after sacrifice. ns means non-significant, * means *p* < 0.05, ** means *p* < 0.01, *** means *p* < 0.001, and **** means *p* < 0.0001.

Our above studies show that **A06** can up-regulate RB gene transcription and translation by unfolding its promoter quadruplex structures, resulting in the inhibition of cancer cell proliferation and metastasis. In order to know whether **A06** could become a potential lead compound for cancer treatment, we carried out further in vivo investigation into its anti-tumor activity. The human cervical cancer xenograft was constructed through the subcutaneous injection of Hela cells into the right shoulder of athymic nude mice at the number of six million cells for each mouse. When the tumor volumes (length × width × width/2) were around 50–100 mm^3^, the nude mice were randomly divided into four groups with five mice in each group, including the high-dose **A06** group (5 mg/kg), low-dose **A06** group (2 mg/kg), saline group as a negative control, and cisplatin group as a positive control (2 mg/kg). The tumor volume and the body weight of the mice were measured every other day after drug treatment. As shown in Figure 8, in terms of tumor volume, the high-dose **A06** group had the smallest tumor volume, followed by the cisplatin group, while the low-dose **A06** group had a smaller tumor volume than the saline group. Tumor volumes were found to be proportional to tumor weights, and the tumor growth inhibition ratio (TGI) was calculated using the equation (1—tumor weight in drug-treated group/tumor weight in saline group) to directly evaluate the tumor inhibition effect. The tumor growth inhibition ratio of the nude mice in the high-dose and low-dose **A06** groups reached 68% and 38%, respectively. For comparison, the tumor growth inhibition ratio for the cisplatin group was about 50%. Apparently, **A06** could effectively inhibit tumor growth in a dose-dependent manner.

**Figure 9:**
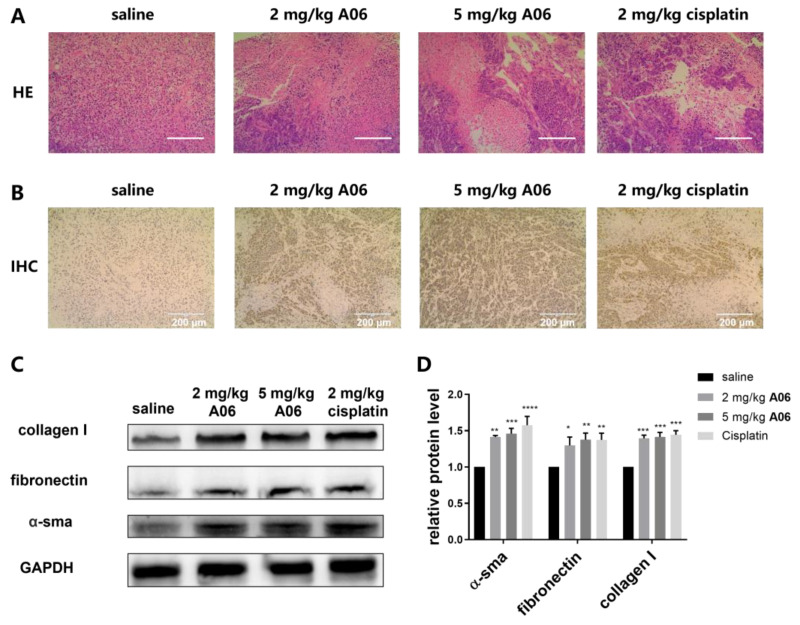
Effect of **A06** on liver and tumor tissues. (**A**) HE-staining images of tumor tissues in different treatment groups (Scale bars: 200 μm). (**B**) Images of tumor immunohistochemistry were obtained with scale bars of 200 μm for images. DAPI is indicated with blue, and RB protein is indicated with brown. (**C**) Western blot for expressions of inflammatory factors in the liver of Hela xenograft model. (**D**) Histogram of gray value analysis for proteins in above (**C**). Experiments were repeated three times, with data shown as mean ± SEM. * means *p* < 0.05, ** means *p* < 0.01, *** means *p* < 0.001, and **** means *p* < 0.0001.

Hematoxylin-eosin staining (HE staining) and immunohistochemistry (IHC) on tumor tissues were carried out to further study the effect of **A06** on tumor inhibition. As shown in Figure 9A, HE staining of tumor tissues showed that the tumor cells of the **A06** group and cisplatin group shrank and became smaller, indicating that **A06** had an inhibitory effect on tumor cells in vivo. In order to confirm that the effect of **A06** on tumor inhibition was relevant to its up-regulation of RB expression, immunohistochemistry was performed to analyze RB expression in the tumor tissues of each group. As shown in Figure 9B, compared with the saline group, the RB protein (brown) expression in the **A06** treatment group and cisplatin group increased in varying degrees. DAPI is indicated in blue, and the RB protein is indicated in brown in the figure. The increased brown area indicated enhanced RB protein expression. The RB protein expression in the **A06** high-dose group was significantly higher than that in the **A06** low-dose group, indicating that **A06** up-regulated RB expression in a dose-dependent manner. In addition, cisplatin could also up-regulate the expression of the RB protein, indicating that the up-regulation of the tumor suppressor gene could play an important role in anti-tumor therapy. In order to evaluate the safety of using **A06** as a potential lead compound, various organs, including the heart, liver, spleen, lung, and kidneys, were weighed with their relative proportions in body weights calculated. As shown in Figure S15A, no significant changes were found in the proportions of these organs in body weights upon drug treatment. The HE-staining experiment showed that drug administrations had no significant detrimental effect on these organs without obvious lesions observed in comparison with the saline group (Figure S15B). The above results show that **A06** had excellent anti-tumor activity in human cervical cancer xenografts without significant toxicity and side effects on essential organs.

The liver is an important organ for drug metabolism, and some toxic metabolites can cause liver damage. Liver fibrosis is the repair of the liver after injury, which is reversible in the short term, but if damage persists, fibrosis becomes irreversible and induces cirrhosis. The toxicity of drugs in the liver can be evaluated by analyzing liver fibrosis. Alpha-smooth muscle actin (α-sma), fibronectin, and collagen I are biomarkers of liver fibrosis [36]. In order to evaluate the potential side effect of **A06** on the liver, the proteins were extracted from liver tissues of various groups for the Western blot analysis of protein expressions of α-sma, fibronectin, and collagen I. As shown in Figure 9C,D, both **A06** and cisplatin promoted the expressions of α-sma, fibronectin, and collagen I in comparison to the saline group, indicating that both compounds could possibly cause liver damage. The fibrotic protein expressions of the cisplatin group were found to be a little higher than those of the **A06** treatment groups, indicating that the hepatotoxicity of compound **A06** was slightly lower than that of cisplatin. Therefore, **A06** could become a promising lead compound for further development without significant toxicity or side effects. It should be noted that although quadruplex-binding ligands have been studied extensively for down-regulating oncogene transcription and translation, as we know, this is the first study for up-regulating a tumor suppressor gene through the destabilization of both the G-quadruplex and i-motif on the gene promoter, which provides a new strategy for innovative anti-cancer drug discovery and development.

## 3. Discussion

I-motif and G-quadruplex are special nucleic acid secondary structures that regulate gene transcription and translation. The ribosomal DNA G-quadruplex ligand Quarfloxin (CX-3543) can inhibit ribosomal transcription, which has shown potent anti-tumor activity in various solid tumors, with its Phase I and Phase II clinical trials finished [37,38]. Our research group has previously reported some small molecules targeting proto-oncogene promoter quadruplex structures for anti-tumor activity. Acridone derivative **B19** has been found to down-regulate c-myc gene transcription and translation by selectively binding to and stabilizing its promoter i-motif structure, resulting in tumor cell apoptosis [17]. Bisacridine derivative **A9** has been found to inhibit SiHa xenograft tumor growth, possibly through its stabilization of both the c-myc promoter G-quadruplex and i-motif [39]. However, previous studies on promoter quadruplex binding ligands focus on down-regulating proto-oncogenes without attention to the up-regulating tumor suppressor gene. It has been found that 125 genes are involved in inducing tumors, of which 70 are tumor suppressor genes, while the others are proto-oncogenes [40]. This suggests that tumor suppressor genes play important roles in regulating tumorigenesis. Current RB-targeting drugs include mainly CDK inhibitors, but it is difficult for these inhibitors to restore RB activity at the protein level due to the problems of drug resistance and very low protein expression levels. Therefore, it is of great significance to develop small-molecule-binding ligands on the RB promoter quadruplex to up-regulate its transcription and translation, producing anti-tumor activity. In addition, the cells in the body are at different phases of cell cycles; therefore, a ligand simultaneously targeting the G-quadruplex (mainly at the S phase) and i-motif (mainly at the G1 phase) could sequentially block the cell cycle and greatly inhibit cell proliferation resulting in enhanced anti-tumor activity with reduced drug administration dosage and low toxicity.

In this study, more than 100 compounds were screened through SPR and MST experiments to obtain the bisacridine derivative **A06**, which had excellent dual binding ability for RB promoter i-motif and G-quadruplex structures. The specific binding and unfolding ability of **A06** to RB promoter quadruplexes was confirmed through TO displacement, CD, and FRET experiments. Quantitative real-time PCR, Western blot, and some other experiments showed that **A06** could effectively up-regulate RB gene transcription and translation without having a significant effect on other genes, which indicated that **A06** regulated RB gene transcription and translation possibly through its selective interaction with RB promoter quadruplex structures. Our cellular-level experiments showed that **A06** had a strong effect on inhibiting the growth and proliferation of tumor cells, inducing the apoptosis of cancer cells, and inhibiting the migration and invasion of tumor cells. The anti-tumor activity of **A06** was further studied using Hela xenografts, which showed that **A06** had strong anti-tumor activity without significant toxicity or side effects. The IHC experiment showed that **A06** promoted RB protein expression in tumor cells, indicating that **A06** inhibited tumor growth, possibly through its up-regulation of RB gene expression. As mentioned earlier, it has been known that RB influences other protein expression mechanisms, usually through the RB-E2F pathway, which regulates a variety of critical tumor processes, including cell cycles, genomic stability, apoptosis, metabolism, differentiation, angiogenesis, immune response, and cell senescence [10]. The primary focus of the present study was to investigate the up-regulation of RB gene transcription and translation by **A06**, leading to increased RB protein expression and producing strong anti-tumor activity. The present study does not provide direct mechanistic evidence to confirm whether **A06**-induced RB expression influences downstream RB-related pathways. It would be interesting to further examine specific downstream targets, such as E2F transcription factors or CDK activity, to confirm RB pathway activation. Additional experiments, including cell cycle analysis, the expression analysis of RB downstream targets (e.g., cyclins, CDKs, E2F-regulated genes), and functional assays assessing RB phosphorylation status, could provide more comprehensive insights into its therapeutic potential, which could be studied in the future. In addition, the development of a small-molecule ligand that specifically targets the RB i-motif will be helpful for the comprehension of the RB i-motif’s biological function. Nevertheless, this is the first study on up-regulating a tumor suppressor gene through the destabilization of both the G-quadruplex and i-motif on the gene promoter, which provides a new strategy for innovative anti-cancer drug discovery and development.

## 4. Materials and Methods

### 4.1. Oligonucleotides and Compounds

All oligonucleotides used in this study were purchased from Sangon (Shanghai, China), with sequences as shown in Table S1. After centrifugation at 5000 rmp for 3 min, the appropriate amount of ddH_2_O was added to make the solution at a concentration of 100 μM. The solution was then stored in a freezer at −20 °C after vortex mixing. Further dilutions to working concentrations were made with relevant buffers as shown in Table 1 [17]:

Compounds for screening have been previously synthesized in our laboratory with structures as shown in Table S2 [17,39,41], including acridone derivatives, bisacridine derivatives, quinoline derivatives, benzothiazole derivatives, fluorescent natural product derivatives, etc. The synthetic method for compound **A06** was shown in Scheme S1. Compounds were dissolved in DMSO at a concentration of 10 mM and stored in a freezer at −20 °C.

### 4.2. Cell Culture

All types of cells, including the cervical adenocarcinoma cell Hela, colorectal adenocarcinoma cell SW480, cervical squamous cell Siha, non-small cell lung cancer cell A549, human colon cancer cell HCT-116, gastric cancer cell HGC-27, liver cancer cell HepG2, and osteosarcoma cell U2OS, were cultured in our laboratory. Cell media, including RPMI-1640, DMEM, and MEM basic media, were mainly used with 10% fetal bovine serum and 1% double antibody added to the medium. HCT-116 and HGC-27 cells were cultured with 10% FBS RPMI-1640 medium. Siha, A549, SW480, HepG2, and U2OS cells were cultured with the DMEM medium containing 10% FBS. Hela cells were cultured using the MEM basic medium containing 10% FBS. The cells were cultured in an ESCO cell incubator at a constant temperature of 37 °C, containing 5% CO_2_.

### 4.3. Surface Plasmon Resonance (SPR) Experiment

The oligomers for RB promoter i-motif and G-quadruplex were used for the experiments with sequences as shown in Table 2. Other oligomers for *braf*, *ACC1*, *ACC2*, *bcl-2*, *RET*, *c-jun*, and *kras* promoter i-motifs had sequences, as shown in Table S1. All buffers for the experiments were prepared with ddH_2_O, which were filtered using the 0.22 μM membrane filter under vacuum. The compounds were dissolved with DMSO at a 10 mM concentration. The content of DMSO in the solution was kept at 0.5%. For insoluble compounds, the DMSO content was increased and kept at 5% with a solvent-corrected measurement procedure used. The principle of half dilution was used to prepare multiple concentration gradients for the experiments. The Biacore X100 intermolecular interaction system with its supporting Biacore-8K Control Software 5.0.18 (Cytiva, Waltham, MA, USA) was used for the experiments. The Biotin-labeled G-rich DNA oligomer was diluted to 0.5 μM with the Tris-HCl buffer at pH 7.4. Biotin-labeled C-rich DNA oligomers were diluted to 0.5 μM with the MES buffer at pH 5.5. The DNA oligomer was denatured by heating at 95 °C for 10 min, slowly cooling to room temperature for the formation of secondary structures through annealing, and storing at 4 °C overnight. The chip channels were immobilized with Streptavidin at first, followed by the annealed DNA oligomers. Ligand solutions were prepared with the running buffer through serial dilutions from stock solutions. The ligands with at least five different concentrations were injected simultaneously at a flow rate of 30 mL/min for 120 s of the association phase, followed by 150 s of the dissociation phase at 25 °C with running buffer (i-motif buffer: 20 mM MES, 100 mM KCl, pH 5.5; G-quadruplex buffer: 50 mM Tris–HCl, 100 mM KCl, pH 7.4). The experimental data were analyzed through curve-fitting with *K*_D_ values determined using the Biacore Insight Evaluation 5.0.18.

### 4.4. Microscale Thermophoresis (MST) Experiment

The MST experiment was performed away from light in order to avoid fluorophore quenching. The 5′-end FAM labeled RB promoter G-quadruplex was purchased from Sangon (China) and was diluted to 2 μM with 50 mM Tris–HCl containing 100 mM KCl at pH 7.4. The 5′-end of the FAM-labeled RB promoter i-motif was purchased from Sangon (China) and was diluted to 2 μM with 20 mM MES containing 100 mM KCl at pH 5.5. The diluted DNA was denatured at 95 °C without light for 10 min, slowly cooled to room temperature for annealing, and stored at 4 °C overnight. The 5′-end FAM-labeled DNA was incubated with compounds of increasing concentrations (diluted at 1:1 from 1000 μM for 16 times) for 10 min at 25 °C. The mixture was transferred into a special capillary tube for MST analysis. The intensities of the LED and laser were set at 20–40%. The *K*_D_ value was determined through the analysis of the data obtained using supporting software NTAnalysis 1.5.41 [42,43].

### 4.5. TO Displacement Experiment

The oligomer for the i-motif was diluted with the BPES buffer at pH 5.5 to 1 μM, while the oligomer for G-quadruplex was diluted with the Tris-HCl buffer at pH 7.4 to 1 μM. The oligomer was annealed at 95 °C for 10 min, cooled to room temperature gradually, and stored at 4 °C for later use. TO dye (1 μL at 200 μM concentration) was added to the DNA solution (100 μL), and the mixture was incubated at room temperature 10 min away from the light. The spectra for the 100 μL mixture with the addition of an increasing amount of the compound at 25 °C were recorded based on fluorescence emission (λex = 480 nm).

TO displacement ratio (%) = (F_0_ − F_C_)/(F_0_ − F_B_) × 100.

F_0_ and Fc were the fluorescence intensity of TO at 536 nm with DNA before and after the addition of the compound, respectively. The fluorescence intensity of TO with the buffer only at 536 nm was recorded as F_B_.

### 4.6. Circular Dichroism (CD) Spectroscopy and CD-Melting Experiment

The oligomer for the i-motif was diluted to 2 μM with the BPES buffer at different pH, while the oligomer for the G-quadruplex was diluted to 2 μM with the Tris-HCl buffer at pH 7.4. The oligomer was heated at 95 °C for 10 min, gradually cooled to room temperature, and then stored at 4 °C overnight. CD spectra from 230 to 350 nm were recorded on a Chirascan^®^ circular dichroism spectrophotometer (Applied Photophysics, Leatherhead, UK) with a 10 mm path length quartz cuvette. The ellipticity change at the highest CD value has been widely used to analyze the thermal stability of the DNA secondary structure [27,44]. The I-motif structure gives two distinct CD bands near 287 nm and 260 nm [45,46], while parallel G-quadruplex shows a positive peak at 262 nm and a negative peak at 240 nm [47]. The CD melting experiment was carried out at a wavelength range of 230–350 nm and temperature range of 25–95 °C, with a heating rate of 2.5 °C/min and scanning interval of 5 °C. The molar ellipticity at 287 nm for the i-motif structure or 260 nm for the G-quadruplex structure was measured. Chirascan 4.1.7 software was used to export experimental data, with a corresponding buffer blank subtracted for all spectra. The graphs were made using GraphPad 7.0 software.

### 4.7. Fluorescence Resonance Energy Transfer (FRET) Experiment

The oligonucleotides with fluorescent 5′-FAM and 3′-TAMRA labeling were diluted to 0.1 μM with a corresponding buffer (i-motif buffer: BPES buffer at different pH; G-quadruplex buffer: Tris-HCl buffer at pH 7.4). After heating at 95 °C for 10 min, the oligonucleotides were annealed gradually and stored in a refrigerator at 4 °C. The spectra from 500 nm to 700 nm were recorded based on fluorescence emission (λex = 480 nm) for the above oligonucleotides upon the addition of an increasing amount of the compound at 25 °C.

### 4.8. NMR Experiment

The oligonucleotide at 600 μM concentration for the RB promoter i-motif was prepared with the BPES buffer at pH 5.5, while the oligonucleotide at a 600 μM concentration for the RB promoter G-quadruplex was prepared with the Tris-HCl buffer at pH 7.4. D_2_O (50 μL) and mixed with the above oligonucleotide (450 μL) to make a total volume of 500 μL. ^1^H NMR titration experiments were performed on Bruker DRX-600 MHz spectrometer at 25 °C. The experimental data were processed using MestReNova 14.0.0 software. The imino proton peaks at 15–16 ppm were analyzed, which are characteristic of the hemiprotonated C-C^+^ base pairs of the i-motif structure [32,33,48].

### 4.9. ESI-MS

The oligomer for the RB promoter i-motif was diluted to 2 μM with the BPES buffer at pH 5.5 or 6.8, while the oligomer for the RB promoter G-quadruplex was diluted to 2 μM with Tris-HCl buffer at pH 7.4. The above oligomer was heated at 95 °C for 10 min, followed by gradual cooling to room temperature. The oligomer was then mixed with two equivalents of the compound, and the mixture was incubated at 4 °C for 12 h, followed by analysis using the LCQ DECA PLUS XP mass spectrometer (Thermo Fisher Scientific, Waltham, MA, USA).

### 4.10. Electrophoretic Mobility Shift (EMSA) Experiment

The oligomer for the RB promoter i-motif was diluted to 2 μM with BPES buffer at pH 5.5, while the oligomer for the RB promoter G-quadruplex was diluted to 2 μM with Tris-HCl buffer at pH 7.4. The above oligomer was heated at 95 °C for 10 min, followed by gradual cooling to room temperature. The oligomer was then mixed with an increasing amount of compound **A06**. Electrophoresis was carried out using 20% acrylamide Native PAGE (pH 7.4 for the RB promoter G-quadruplex and pH 5.5 for the RB promoter i-motif) with 1x Tris-borate-EDTA (TBE) as the running buffer (pH 7.0 for the RB promoter G-quadruplex and pH 6.0 for the RB promoter i-motif) at 80 V for 5 h at 4 °C. The gels were then silver-stained and photographed.

### 4.11. Ultraviolet–Visible (UV) Spectroscopy

The oligomer for the RB promoter i-motif was diluted to 500 μM with the BPES buffer at pH 5.5, while the oligomer for the RB promoter G-quadruplex was diluted to 500 μM with Tris-HCl buffer at pH 7.4. The above oligomer was heated at 95 °C for 10 min, followed by gradual cooling to room temperature, and stored at 4 °C for later use. Compound **A06** (10 μM) was prepared with the corresponding buffer, and its UV absorption spectrum was analyzed using a UV spectrophotometer at the scanning range of 350–550 nm. The oligomer for the RB promoter i-motif or G-quadruplex was added dropwise to increase the final concentration by 1 μM with the mixture incubated for 10 min for each following UV scanning. The percentage decrease or increase in absorbance of the compound with the increasing concentration of DNA was evaluated using the following equation:

H (%) = (A − A_0_)/A_0_ × 100

A_0_ and A are the absorbance data of the compound without and with increasing the concentration of DNA, respectively [49].

### 4.12. Dual-Luciferase Reporter Assay

The luciferase pGL-3 basic plasmids with the RB promoter wild type, deleted sequence, and mutant sequence were constructed, as shown in Table S3. The cervical adenocarcinoma cell Hela (100 μL) was added to a 96-well plate at a concentration of about 5 × 10^3^ cells/well. After incubation for 24 h, 100 ng if the constructed recombinant plasmid pGL-Wt RB (or pGL-Del RB, or pGL-Mut RB) with 100 ng of the Renilla control plasmid pRL-TK was transfected into Hela cells, and each group was made in triplicate. After incubation for 24 h, the medium was replaced with an increasing concentration of the compound to make a total volume of 100 μL per well, and the resulting mixture was further incubated for 48 h. The expression of firefly luciferase was analyzed and normalized to the Renilla luciferase signal. The graphs were made using GraphPad 7.0 software.

### 4.13. Reverse Transcription–Quantitative Polymerase Chain Reaction (RT-qPCR)

Total RNA was isolated from Hela cells treated with the compound for 24 h using the AG RNAex Pro Reagent (Accurate Biology, Changsha, China, #AG21101). Total RNA (1 μg) was used as a template for reverse transcription using a PrimeScript™ RT reagent Kit with gDNA Eraser (TAKARA, Osaka, Japan, #RR047A) following the manufacturer’s protocol. Genomic DNA was removed with gDNA Eraser at 42 °C for 2 min and then subjected to cDNA synthesis in a 20 μL volume. The mixtures were incubated at 37 °C for 15 min of reverse transcription and then at 85 °C for 5 sec. PCR was performed in duplicate with 10 μM primers (Table S4) and 2× SYBR^®^ Green Pro Taq HS Premix (GenStar, Guangzhou, China) with 1 μL cDNA template using the LightCycler^®^480 system (Roche, Basel, Switzerland). PCR was performed at 95 °C for 5 min, and then through 40 cycles at 95 °C for 15 s and 60 °C for 1 min, followed by a dissociation stage at 95 °C for 5 s, 65 °C for 1 min, and then 40 °C for 30 s for melting curve analysis. Each group was made in triplicate.

### 4.14. Western Blot

Hela cells treated with compounds for 24 h were collected and lysed in the RIPA lysis buffer (Bioteke, Wuxi, China). The protein concentration was determined with the BCA protein assay kit (Thermo Fisher Scientific, Waltham, MA, USA). An equal amount of protein (20 μg) was resolved using 10% SDS-PAGE and then transferred to the 0.22 μm PVDF membrane. The blots were blocked for 15 min with 5% BSA, and the target protein bands were cut according to protein markers, which were then probed with primary antibodies (1:1000). After four washes in TBST, the membranes were incubated with corresponding secondary antibodies (1:3000). Blots were visualized using chemiluminescence, and blot images were acquired using the Tanon-4200SF gel imaging system (Tanon Science & Technology Co., Ltd., Shanghai, China).

### 4.15. Immunofluorescence

Hela cells grown on glass coverslips were treated with increasing concentrations of the compound for 48 h, which were then fixed with 4% formaldehyde for 30 min at 37 °C, permeabilized with 0.1% Triton-X100/PBS (phosphate-buffer saline), and blocked with 5% BSA. For immunolabeling experiments, cells were incubated with the RB antibody at 4 °C overnight. Then, the cells were washed with PBS three times and incubated with the anti-rabbit Alexa 488-conjugated antibody (#A21206, Life Technology, Carlsbad, CA, USA) at 37 °C for 30 min. Nuclei were counter-stained with DAPI (Invitrogen, Carlsbad, CA, USA) at room temperature for 20 min. Then, the cells were washed with PBS and analyzed using an FV3000 laser scanning ultra-high-resolution microscope (Olympus, Tokyo, Japan) for the expression of RB.

### 4.16. Cytotoxic Assay

In 96-well microplates, 5 × 10^3^ cells were inoculated into each well and cultured overnight. Cells were incubated with various concentrations of compounds (0–50 μM) for 48 h. Then, 100 μL of the 0.5 mg/mL methyl thiazolyl tetrazolium (MTT) solution was added to each well, and the cells were further incubated for 4 h. Next, the medium was discarded, and dimethyl sulfoxide (DMSO) was added with 100 μL per well to each well to dissolve the formazan dye. A microplate reader (Bio-Tek, Winooski, VT, USA) was used to measure the absorbance at 570 nm. All experiments were parallel and performed in triplicate, and the IC_50_ values were determined through the mean OD values of the triplicate tests versus the drug concentration curves.

### 4.17. Colony Formation Assay

Hela cells were inoculated in a 6-well plate and continuously cultivated for 24 h. The cells were exposed to compounds at increasing concentrations and DMSO as a control at 37 °C in a humidified atmosphere with 5% CO_2_. The fresh drug-containing MEM basic medium was replaced every three days, during which the morphology and growth of the cells were observed and recorded under a microscope. After nine days of cell culture, the cells were fixed with methyl alcohol and dyed with crystal violet. The pictures were taken using a cell imager.

### 4.18. FITC Annexin V/PI Cell Apoptosis Analysis

FITC Annexin V/PI cell apoptosis analysis was performed using the Annexin V-FITC/PI Apoptosis Kit (#AP101-100-kit, Multi Sciences, Hangzhou, China) following the manufacturer’s protocol for the kit. The emission fluorescence was quantified using Epics Elite flow cytometry (Beckman Coulter, Brea, CA, USA). For each analysis, 10,000 events were collected.

### 4.19. Hoechst Staining

Hela cells were inoculated in a 6-well plate (2 × 10^5^ cells per well) and cultured in a cell incubator for 24 h. Then, the cells were incubated with increasing concentrations of compounds for 48 h. The medium was discarded, and the cells were fixed with paraformaldehyde for 30 min. After washing the cells with PBS, Hoechst 33342 (Beyotime, Shanghai, China) was added to stain the cells for 20 min. The cells were photographed and analyzed using a cell imaging system.

### 4.20. Cell Scrape Assay

Hela cells were seeded on 6-well culture plates (1,000,000/well) at 37 °C in a humidified atmosphere with 5% CO_2_. After 24 h preculture, a cross-shaped scrape was made through monolayer Hela cells using a plastic pipet’s tip. The cells were washed three times with a serum-free medium and incubated with increasing concentrations of the compound for 48 h. Several wounded areas were observed and photographed using microscopy. For ease of observation, the edges of the cells were marked with lines.

### 4.21. Transwell Assay

Matrigel was diluted with a serum-free medium at a ratio of 1:8, which was added to 8 μm transwell inserts in 24-well plates and solidified in a 37 °C incubator for 4–6 h for the formation of a thin gel layer. Then, Hela cells with 200 μL of the serum-free medium were plated on the transwell insert, and 400 μL of the medium containing 20% FBS was added to the lower chamber. Compound **A06** at increasing concentrations was added to transwell inserts. After 48 h, the medium within the transwell inserts was carefully removed. The cells on the membrane side in contact with the lower chamber were fixed with 4% paraformaldehyde for 20 min. The cells were stained with 1% crystal violet dye for 20 min after washing with PBS. The invasive cells were observed and photographed using the EVOS FL Auto cell imaging system (Thermo Fisher Scientific, Waltham, MA, USA).

### 4.22. Evaluation of In Vivo Anti-Tumor Activity

Female BALB/c nude mice (4 weeks old) were purchased from and housed at the Guangdong Medical Laboratory Animal Center (Guangzhou, China). The mice were maintained in pathogen-free conditions for 12 h and given food and water in a light–dark cycle of 24 ± 1 °C and 60–70% humidity. All procedures were approved by the Sun Yat-sen University Animal Care and Use Committee and complied with legal provisions and national guidelines on the care and maintenance of laboratory animals (Approval No. SYSU-IACUC-2022-001720). Approval date: 10 November 2022. Effective date: 10 November 2022 to present. Each mouse was injected subcutaneously with 6 × 10^6^ Hela cells using a 22-gauge needle syringe.

About 8 days after tumor implantation, significant tumor tissue with a volume of 50–100 mm^3^ could be observed. The mice were randomly divided into four groups, including the normal saline solvent control group, 5 mg/kg **A06** hydrochloride high-dose administration group, 2 mg/kg **A06** hydrochloride low-dose administration group, and 2 mg/kg cisplatin group. The drug was administered every other day by intraperitoneal injection. The tumor volume and the body weight of the mice were measured every other day after drug treatment.

At the end of the experiment, the nude mice were euthanized, and five vital organs of the nude mice, including the heart, liver, spleen, lung, and kidney, as well as tumor tissues, were removed. The organs and tumor tissues were weighed and recorded and then cut into two parts, stored at −80 °C and fixed with paraformaldehyde, respectively. The inhibition ratio (IR) was calculated according to the following formula: IR = (1 − mean tumor weight of the experimental group/mean tumor weight of the control group) × 100%. The fixed tissues and organs were sent to Sevier Bio for IHC- and HE-stained sections. The expressions of α-sma, collagen I, and fibronectin proteins in liver tissues were analyzed using Western blot.

### 4.23. Statistical Analysis

Data were expressed as the mean ± SEM. Data between the two groups were analyzed with one-way ANOVA or unpaired Student’s *t*-tests using GraphPad Prism (GraphPad Software Inc., La Jolla, CA, USA). A * *p*-value of ≤0.05 was considered statistically significant.

## Figures and Tables

**Table 1:** Different buffers used in this study.

Buffer	Ingredients
MES buffer	20 mM MES, 100 mM KCl, pH 5.5
BPES buffer	30 mM (KH_2_PO_4_, K_2_HPO_4_), 1 mM EDTA, and 100 mM KCl
Tris–HCl buffer	50 mM Tris–HCl, 100 mM KCl, pH 7.4

**Table 2:** Oligonucleotides used in SPR experiment.

Oligomer	Sequence
RB IM for RB promoter i-motif	5′-GCCGCCCAAAACCCCCCG-3′
RB G4 for RB promoter G-quadruplex	5′-CGGGGGGTTTTGGGCGGC-3′

## Data Availability

The data presented in this study are available in both the article and Supplementary Materials.

## Supplementary Materials

The following supporting information can be downloaded at: https://www.mdpi.com/article/10.3390/ijms26041417/s1.
