# Supplementary material for: Selective Up-Regulation of Tumor Suppressor Gene Retinoblastoma by Bisacridine Derivative Through Gene Promoter Quadruplex Structures for Cancer Treatment
Source: Int J Mol Sci. 2025 Feb 7;26(4):1417. doi: 10.3390/ijms26041417 (PMC11855212; doi:10.3390/ijms26041417)
Supplement: Supplementary file 1 [file ijms-26-01417-s001.zip › ijms-3426329-supplementary.pdf]

Supplementary Materials

Selective Up-Regulation of Tumor Suppressor Gene Retinoblastoma by Bisacridine Derivative Through Gene Promoter Quadruplex Structures for Cancer Treatment

Xiaomin Lin †, Jiahui Zhang †, Jihai Liang, Dongsheng Ji, Zhi-Shu Huang and Ding Li *

School of Pharmaceutical Sciences, Sun Yat-Sen University, Guangzhou University City, Guangzhou 510006, China

* Correspondence: liding@mail.sysu.edu.cn; Tel: +86-20-3994-3058

† These authors contributed equally to this work.

Table of Contents

**Figure S1.** CD experiment showed that RB gene promoter could form i-motif and G-quadruplex structures	**S3**
**Figure S2.** K_D values for binding of compounds to RB quadruplexes were determined through MST experiment	**S3**
**Figure S3.** Effects of **A06** and **A02** on RB promoter quadruplex structures were studied by using CD and CD-melting experiments	**S4**
**Figure S4.** Fluorescence response experiments were carried out to study the effects of compounds on the formation of RB promoter quadruplex structures	**S5**
**Figure S5.** SPR experiment was performed for binding of **A06** to other gene promoter quadruplex structures	**S6**
**Figure S6.** TO displacement was performed for binding of **A06** to quadruplexes	**S8**
**Figure S7.** CD experiment was performed for interaction of **A06** with other gene quadruplex structures	**S9**
**Figure S8.** Fluorescence response experiments were carried out to study the effect of **A06** on other gene quadruplex structures	**S10**
**Figure S9.** UV titration experiment was performed to study the interaction of **A06** with RB promoter quadruplex structures	**S10**
**Figure S10.** EMSA experiment was performed with silver staining for the effect of **A06** on RB promoter quadruplex structures	**S11**
**Figure S11.** ESI-MS experiment was performed for binding interaction of **A06** with RB	**S12**

quadruplex structures	
**Figure S12.** The effect of **A06** on RB gene transcription and translation	**S13**
**Figure S13.** Immunofluorescence image of RB protein expressed in Hela cells incubated with different concentrations of **A06** for 48 h	**S14**
**Figure S14.** The anti-apoptotic effect of compound **A06** on different cancer cells	**S15**
**Figure S15.** Comparison of vital organs for mice in different treatment groups	**S16**
**Scheme S1.** Synthetic pathway for bisacridine derivative **A06** and **A06·HCl**	**S16**
**Table S1.** Oligonucleotides used in this study	**S16**
**Table S2.** Equilibrium binding constants (K_D) determined by using SPR	**S18**
**Table S3.** The oligonucleotides for wild type or deleted RB promoter used in pGL-3 Basic plasmids	**S21**
**Table S1.** Primers used for qRT-PCR	**S22**
**Table S2.** IC_{50} (μM) values were determined for effects of **A06** and **A02** on various tumor cells by using MTT assay	**S23**
^{1}H NMR and ^{13}C NMR spectra of bisacridine derivatives	**S24**

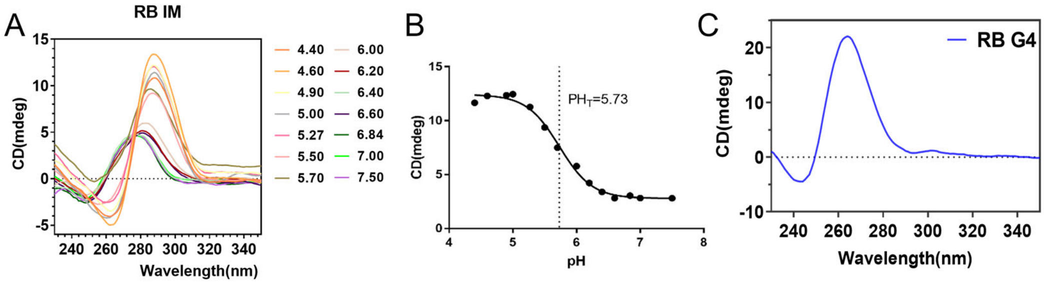

Figure S1. CD experiment showed that RB gene promoter could form i-motif and G-quadruplex structures. (A) CD spectrum of RB promoter i-motif in BPES buffer at different pH. (B) CD spectrum value at 288 nm for RB promoter i-motif against pH was used to determine pH_T through curve fitting. (C) CD spectrum of RB promoter G-quadruplex in Tris-HCl buffer at pH 7.4.

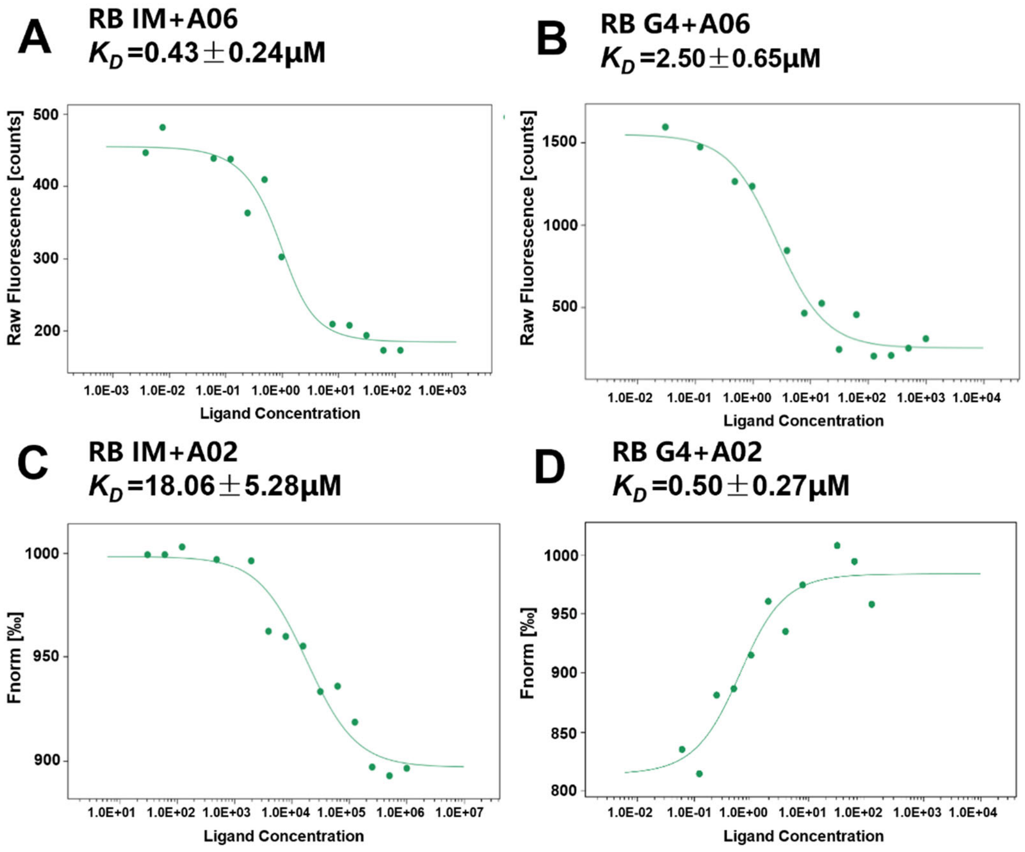

Figure S2. K_D values for binding of compounds to RB quadruplexes were determined through MST experiment. (A) K_D value for binding of **A06** to RB promoter i-motif was determined to be 0.43±0.24 μM in MES buffer at pH 5.5. (B) K_D value for binding of **A06** to RB promoter G-quadruplex was determined to be 2.50±0.65 μM in Tris-HCl buffer at pH 7.4. (C) K_D value for binding of **A02** to RB promoter i-motif was determined to be 18.06±5.28 μM in MES buffer at pH 5.5. (D) K_D value for binding of **A02** to RB promoter G-quadruplex was determined to be 0.50±0.27 μM in Tris-HCl buffer at pH 7.4.

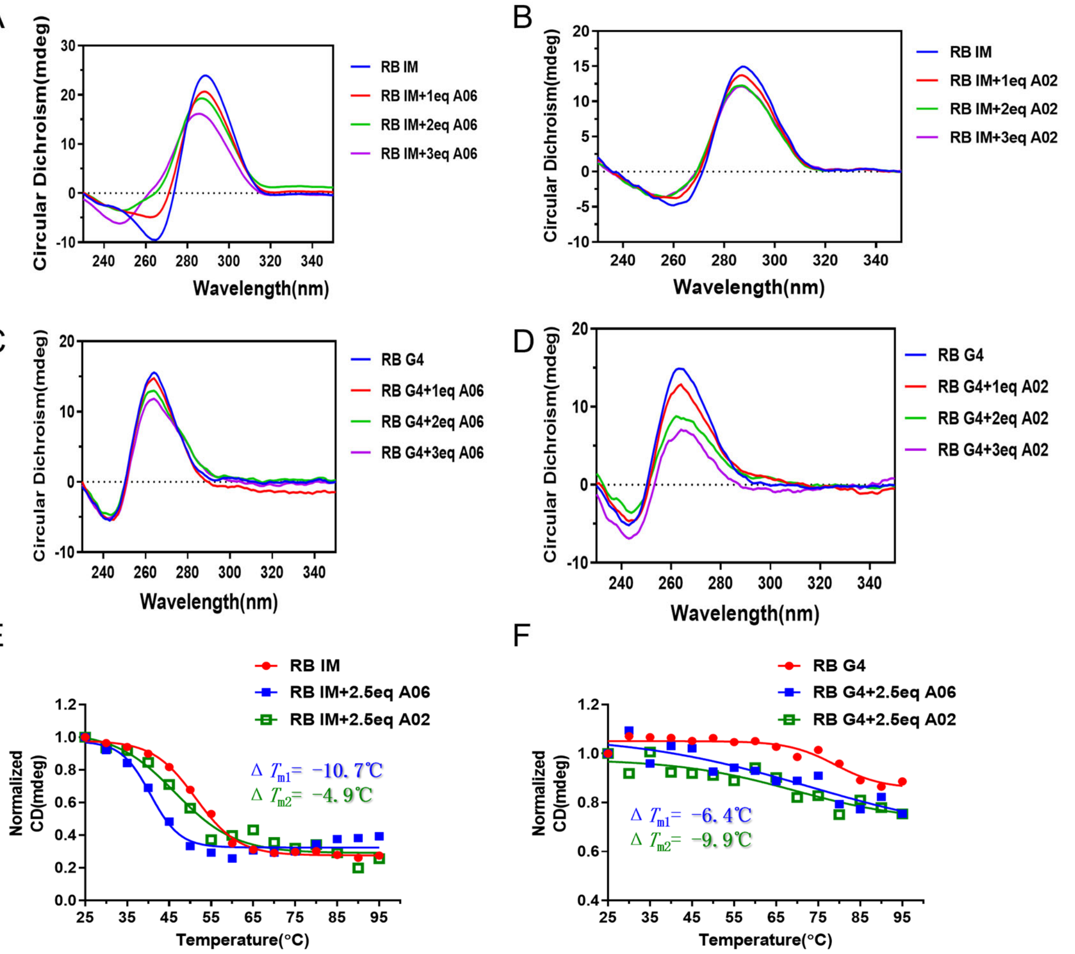

Figure S3. Effects of **A06** and **A02** on RB promoter quadruplex structures were studied by using CD and CD-melting experiments. (A) CD experiment was performed for RB promoter i-motif structure with addition of increasing amount of **A06** in BPES buffer at pH 5.5. (B) CD experiment was performed for RB promoter i-motif structure with addition of increasing amount of **A02** in BPES buffer at pH 5.5. (C) CD experiment was performed for RB promoter G-quadruplex structure with addition of increasing amount of **A06** in Tris-HCl buffer at pH 7.4. (D) CD experiment was performed for RB promoter G-quadruplex structure with addition of increasing amount of **A02** in Tris-HCl buffer at pH 7.4. (E) CD-melting experiment was performed for RB promoter i-motif structure with addition of **A06** or **A02**. The T_m values were determined to be 40.6 °C for RB promoter i-motif with **A06** and 46.4 °C for RB promoter i-motif with **A02**. ΔT_m values were determined to be -10.7 °C for **A06** and -4.9 °C for **A02** respectively. (F) CD-melting experiment was performed for RB promoter G-quadruplex structure with addition of **A06** or **A02**. The T_m values were determined to be 72.7 °C for RB promoter G-quadruplex with **A06** and 69.2 °C for RB promoter G-quadruplex with **A02**. ΔT_m values were determined to be -6.4 °C for **A06** and -9.9 °C for **A02** respectively.

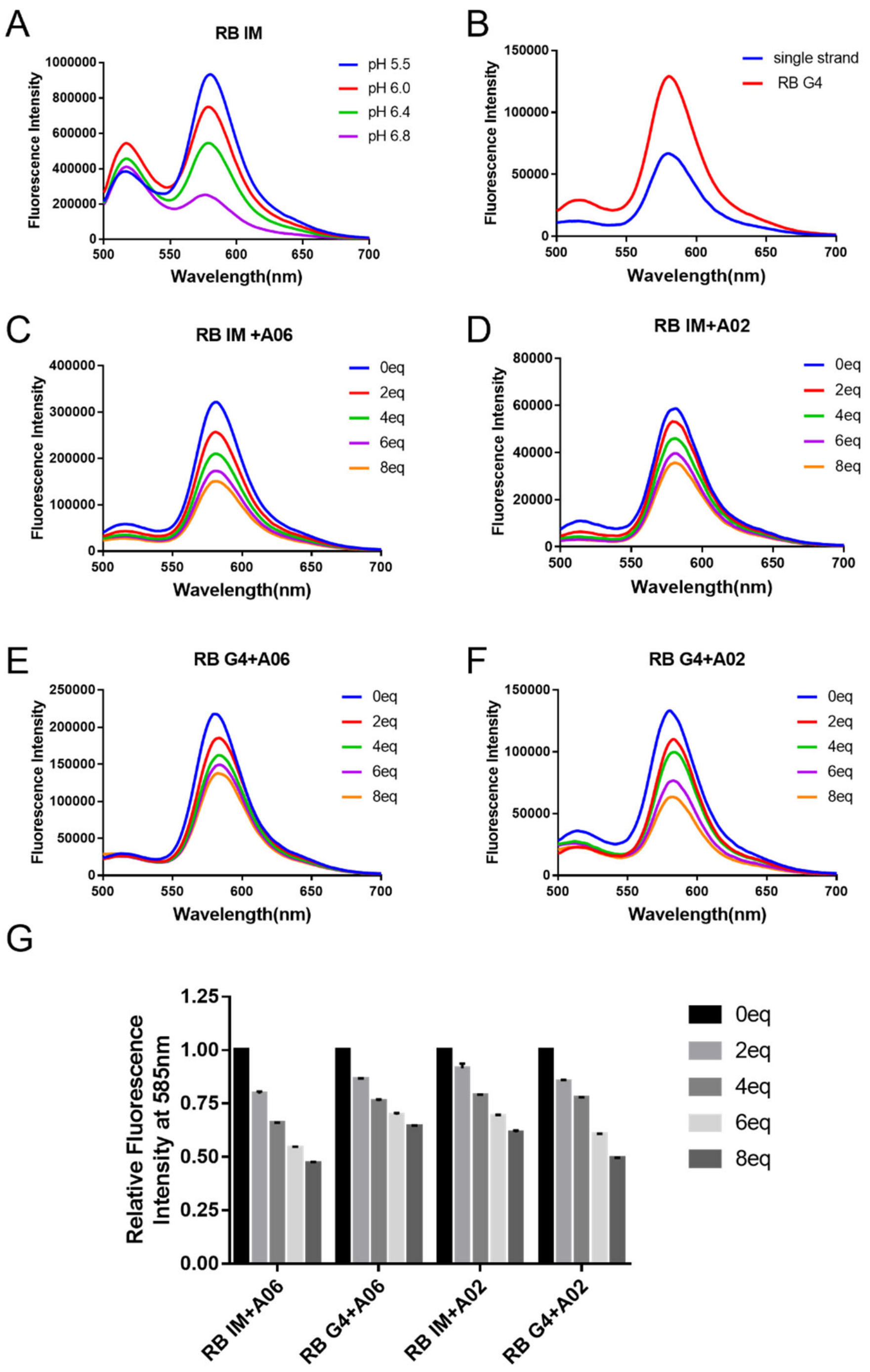

Figure S4. Fluorescence response experiments were carried out to study the effects of compounds on the formation of RB promoter quadruplex structures. (A) Fluorescence response curves of double-labeled RB promoter i-motif sequences annealed with BPES buffer at different pH. (B) Fluorescence response curves of RB promoter G-quadruplex sequences annealed and unannealed in Tris-HCl buffer at pH 7.4. (C) The fluorescence response curves were measured with double-labeled RB promoter i-motif after annealing in BPES buffer at pH 5.5 upon incubation with increasing concentration of **A06** for 10 min. (D) The fluorescence response curves were measured with double-labeled RB promoter i-motif after annealing in BPES buffer

at pH 5.5 upon incubation with increasing concentration of **A02** for 10 min. (E) The fluorescence response curves were measured with double-labeled RB promoter G-quadruplex after annealing in Tris-HCl buffer at pH 7.4 upon incubation with increasing concentration of **A06** for 10 min. (F) The fluorescence response curves were measured with double-labeled RB promoter G-quadruplex after annealing in Tris-HCl buffer at pH 7.4 upon incubation with increasing concentration of **A02** for 10 min. (G) The relative fluorescence intensity at 585 nm was measured for RB promoter quadruplex structures with the addition of 0, 2, 4, 6 and 8 eq compounds. The experimental results were normalized and graphed, with all experiments repeated for three times in parallel. The data were expressed as mean ± SEM.

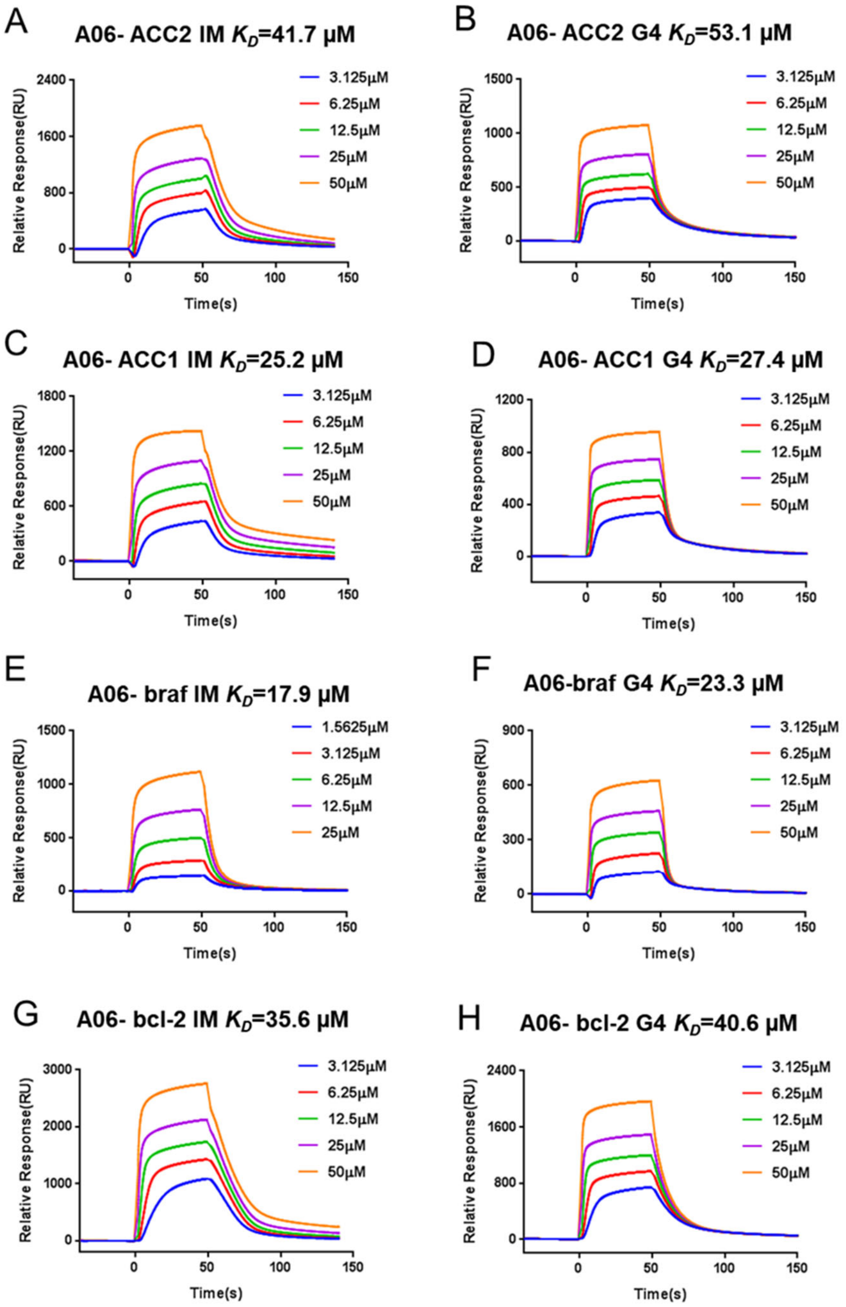

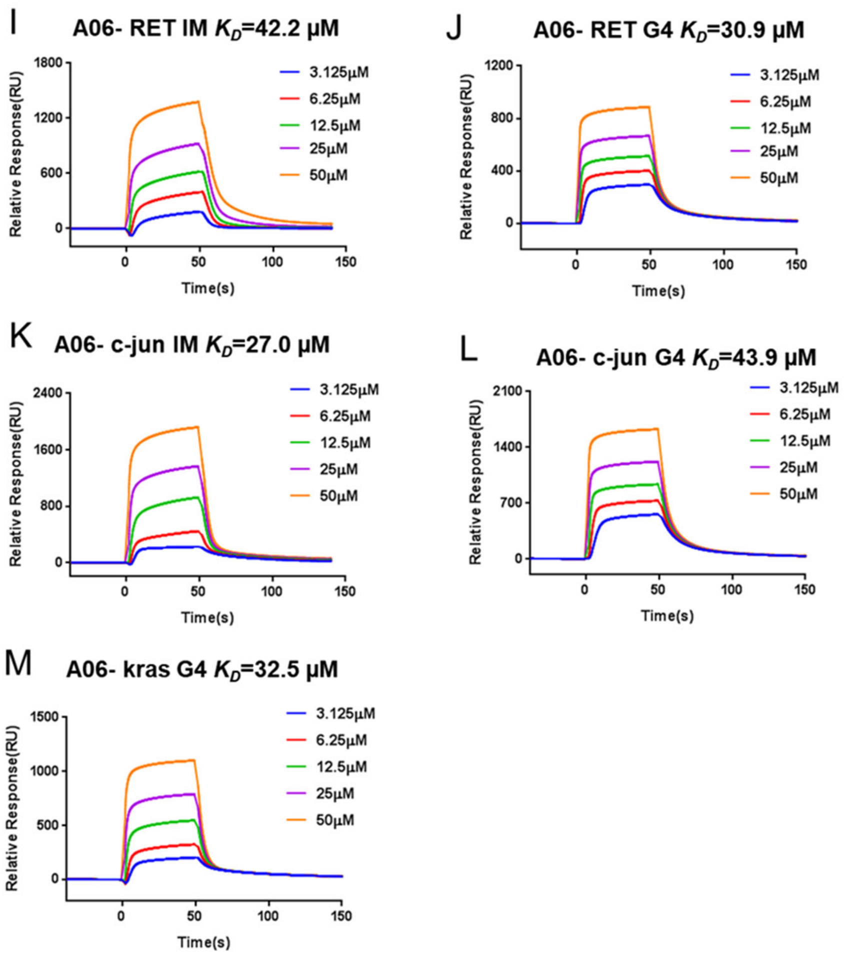

Figure S5. SPR experiment was performed for binding of **A06** to other gene promoter quadruplex structures. (A) K_D value for binding of **A06** with ACC2 promoter i-motif was determined to be 41.7 μM in MES buffer at pH 5.5. (B) K_D value for binding of **A06** with ACC2 promoter G-quadruplex was determined to be 53.1 μM in Tris-HCl buffer at pH 7.4. (C) K_D value for binding of **A06** with ACC1 promoter i-motif was determined to be 25.2 μM in MES buffer at pH 5.5. (D) K_D value for binding of **A06** with ACC1 promoter G-quadruplex was determined to be 27.4 μM in Tris-HCl buffer at pH 7.4. (E) K_D value for binding of **A06** with BRAF promoter i-motif was determined to be 17.9 μM in MES buffer at pH 5.5. (F) K_D value for binding of **A06** with BRAF promoter G-quadruplex was determined to be 23.3 μM in Tris-HCl buffer at pH 7.4. (G) K_D value for binding of **A06** with bcl-2 promoter i-motif was determined to be 35.6 μM in MES buffer at pH 5.5. (H) K_D value for binding of **A06** with bcl-2 promoter G-quadruplex was determined to be 40.6 μM in Tris-HCl buffer at pH 7.4. (I) K_D value for binding of **A06** with RET promoter i-motif was determined to be 42.2 μM in MES buffer at pH 5.5. (J) K_D value for binding of **A06** with RET promoter G-quadruplex was determined to be 30.9 μM in Tris-HCl buffer at pH 7.4. (K) K_D value for binding of **A06** with c-jun promoter i-motif was determined to be 27.0 μM in MES buffer at pH 5.5. (L) K_D value for binding of **A06** with c-jun promoter G-quadruplex

was determined to be 43.9 μM in Tris-HCl buffer at pH 7.4. (M) K_D value for binding of **A06** with KRAS promoter G-quadruplex was determined to be 32.5 μM in Tris-HCl buffer at pH 7.4.

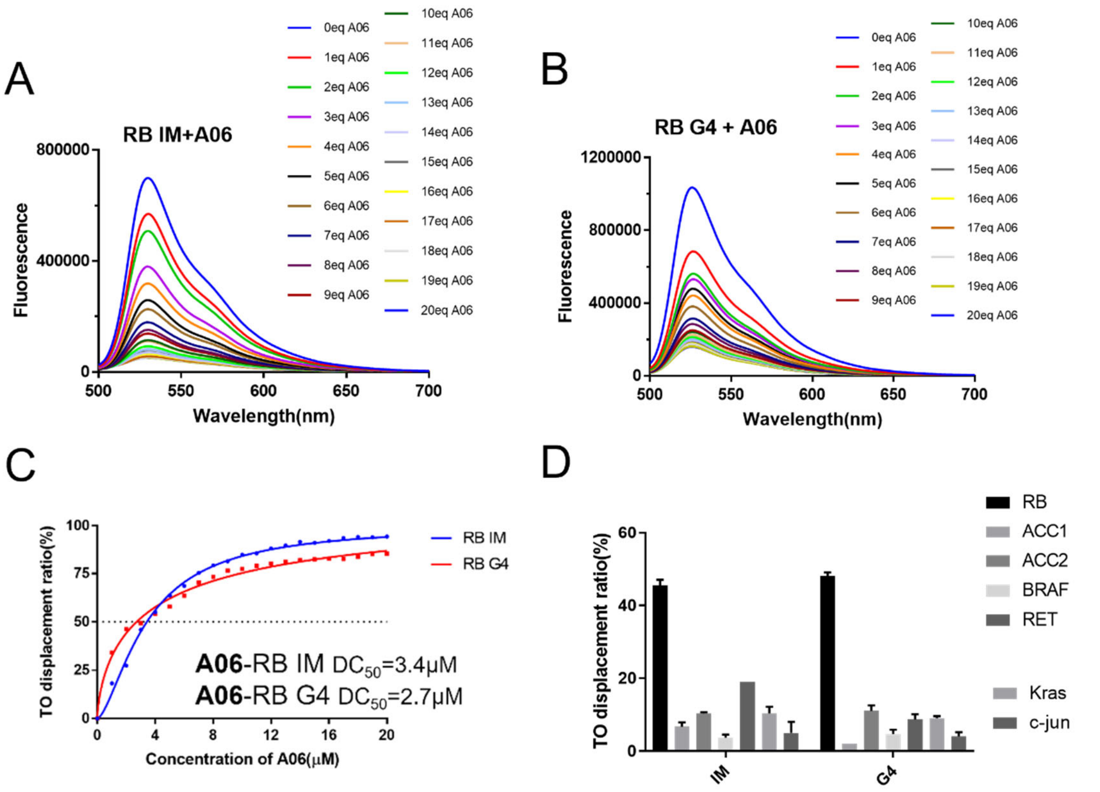

Figure S6. TO displacement was performed for binding of **A06** to quadruplexes. (A) Fluorescence spectra of TO displacement experiment for RB promoter i-motif (1 μM) with increasing concentration of **A06**. (B) Fluorescence spectra of TO displacement experiment for RB promoter G-quadruplex (1 μM) with increasing concentration of **A06**. (C) The plots of displacement ratio for RB promoter i-motif/G-quadruplex against concentration of **A06**, with DC_{50} values determined to be 3.4 μM for RB promoter i-motif and 2.7 μM for RB promoter G-quadruplex. (D) Histogram of TO displacement ratio of 3 μM **A06** for different gene quadruplexes with λex at 480 nm and λem at 530 nm. All experiments were repeated for three times in parallel. The data were expressed as mean ± SEM.

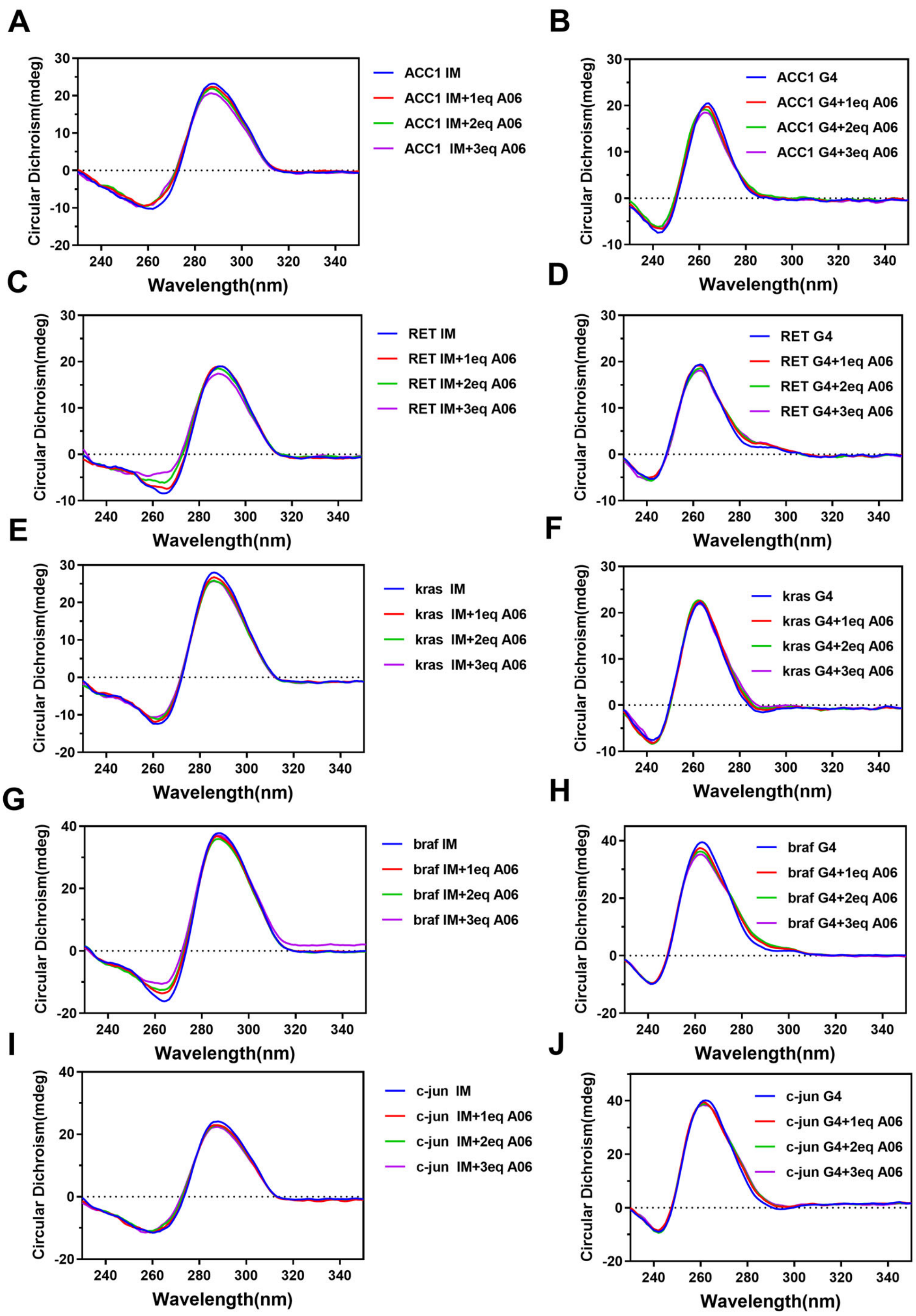

Figure S7. CD experiment was performed for interaction of **A06** with other gene quadruplex structures. (A) CD spectrum of ACC1 promoter i-motif structure with increasing amount of **A06** in BPES buffer at pH 5.5. (B) CD spectrum of ACC1 promoter G-quadruplex structure with increasing amount of **A06** in Tris-HCl buffer at pH 7.4. (C) CD spectrum of RET promoter i-motif structure with increasing amount of **A06** in BPES buffer at pH 5.5. (D) CD spectrum of RET promoter G-quadruplex structure with increasing amount of **A06** in Tris-HCl buffer at pH 7.4. (E) CD spectrum of KRAS promoter i-motif structure with increasing amount of **A06** in BPES buffer at pH 5.5. (F) CD spectrum of KRAS promoter G-quadruplex structure with increasing

amount of **A06** in Tris-HCl buffer at pH 7.4. (G) CD spectrum of BRAF promoter i-motif structure with increasing amount of **A06** in BPES buffer at pH 5.5. (H) CD spectrum of BRAF promoter G-quadruplex structure with increasing amount of **A06** in Tris-HCl buffer at pH 7.4. (I) CD spectrum of c-jun promoter i-motif structure with increasing amount of **A06** in BPES buffer at pH 5.5. (J) CD spectrum of c-jun promoter G-quadruplex structure with increasing amount of **A06** in Tris-HCl buffer at pH 7.4.

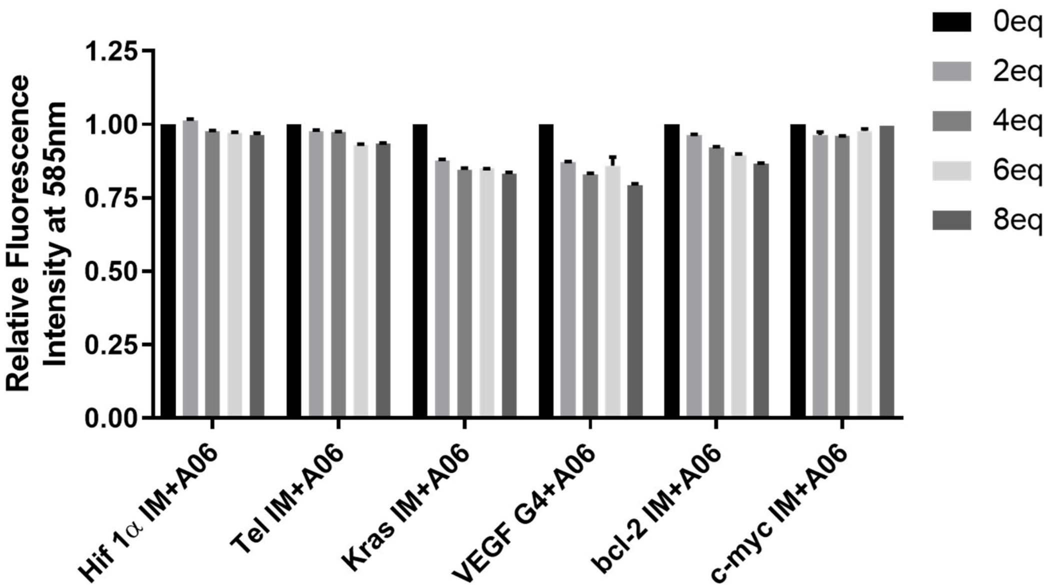

Figure S8. Fluorescence response experiments were carried out to study the effect of **A06** on other gene quadruplex structures. The relative fluorescence intensity at 585nm of other gene quadruplex structures were measured after addition of 0, 2, 4, 6, and 8 eq **A06**. The experimental results were normalized and graphed. All experiments were repeated for three times in parallel, with the data expressed as mean ± SEM.

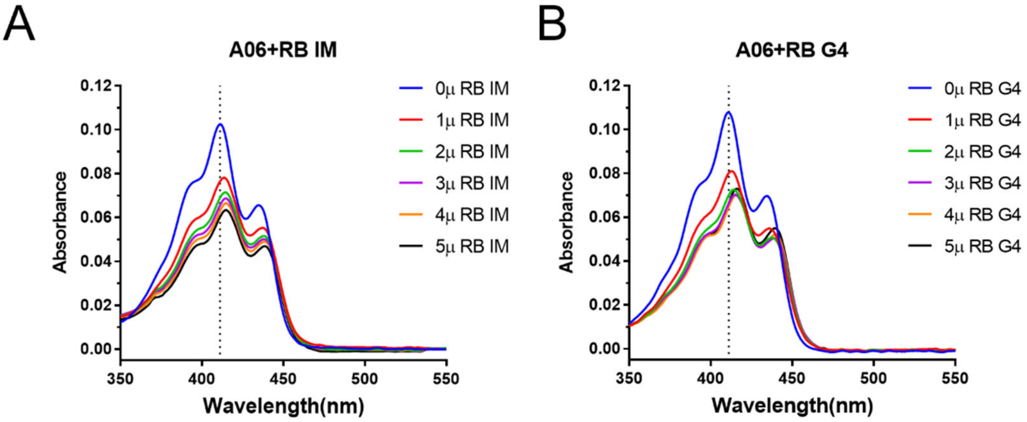

Figure S9. UV titration experiment was performed to study the interaction of **A06** with RB

promoter quadruplex structures. (A) UV titration experiment for incubation of 10 μM **A06** with increasing concentration of RB promoter i-motif in BPES buffer at pH 5.5. (B) UV titration experiment for incubation of 10 μM **A06** with increasing concentration of RB promoter G-quadruplex in Tris-HCl buffer at pH 7.4.

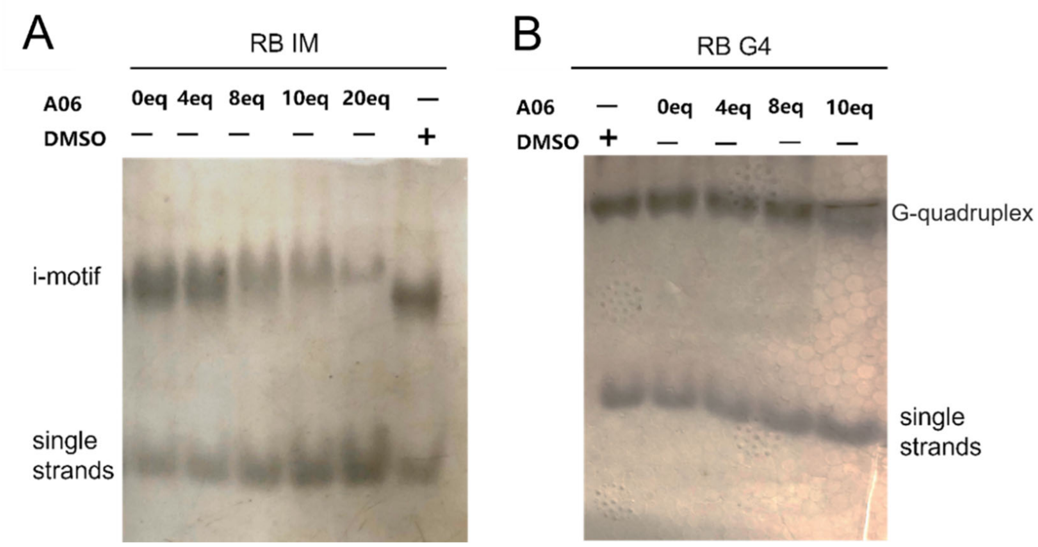

Figure S10. EMSA experiment was performed with silver staining for the effect of **A06** on RB promoter quadruplex structures. (A) Compound **A06** destabilized RB promoter i-motif structure in BPES buffer at pH 5.5. Channels from left to right were follow: RB promoter i-motif, RB promoter i-motif + 4 eq **A06**, RB promoter i-motif + 8 eq **A06**, RB promoter i-motif + 10 eq **A06**, RB promoter i-motif + 20 eq **A06**, and RB promoter i-motif + 20 eq DMSO. (B) Compound **A06** destabilized RB promoter G-quadruplex structure in Tris-HCl buffer at pH 7.4, and the channels from left to right were follow: RB promoter G-quadruplex + 20 eq DMSO, RB promoter G-quadruplex, RB promoter G-quadruplex + 4 eq **A06**, RB promoter G-quadruplex + 8 eq **A06**, and RB promoter G-quadruplex +10 eq **A06**.

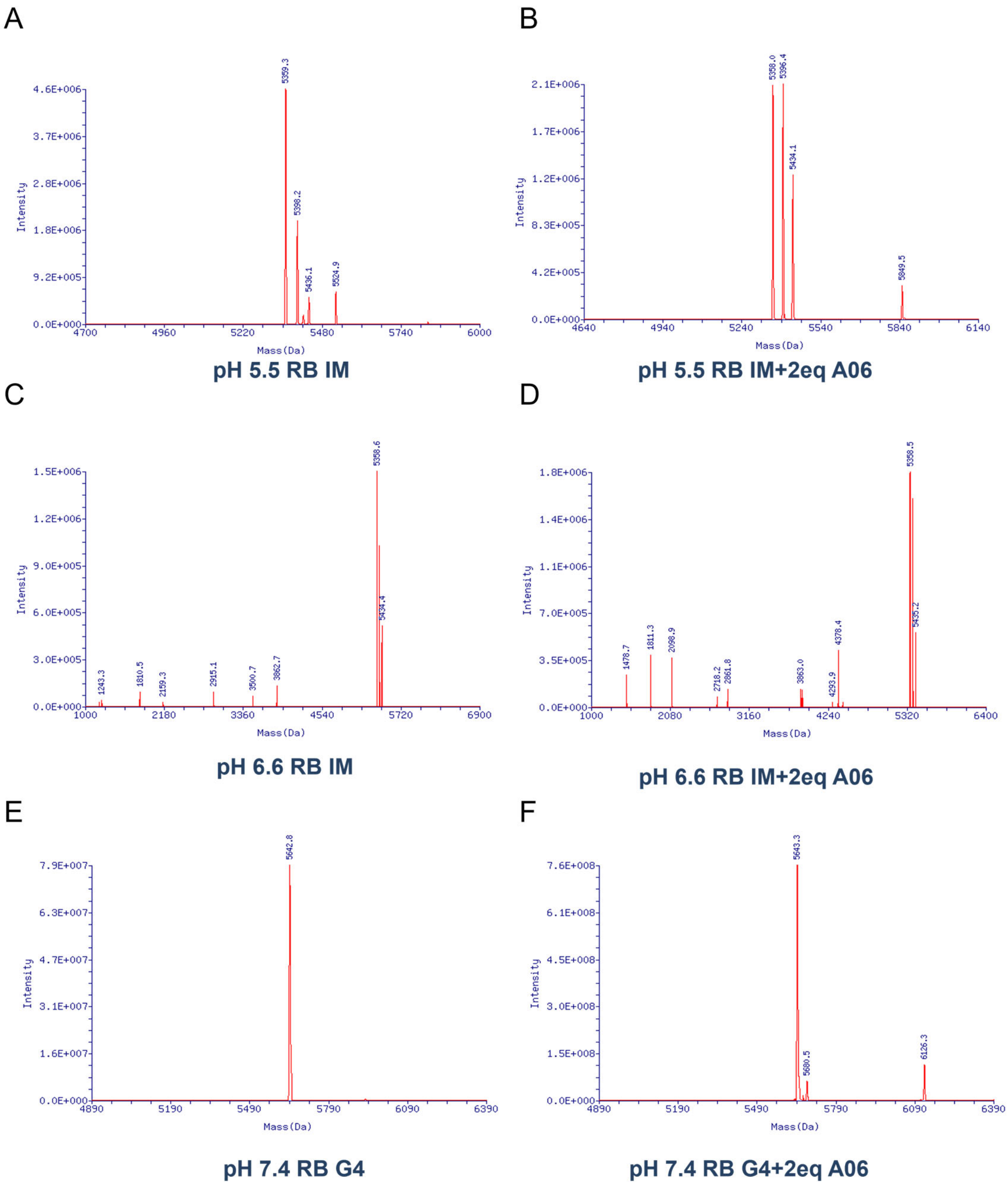

Figure S11. ESI-MS experiment was performed for binding interaction of **A06** with RB quadruplex structures. (A) Mass spectrum of RB promoter i-motif annealed in BPES buffer at pH 5.5. (B) Mass spectrum for binding of 2 eq **A06** with RB promoter i-motif annealed in BPES buffer at pH 5.5. (C) Mass spectrum of RB promoter i-motif annealed in BPES buffer at pH 6.6. (D) Mass spectrum of RB promoter i-motif annealed in BPES buffer at pH 6.6 with addition of 2 eq **A06**. (E) Mass spectrum of RB promoter G-quadruplex annealed in Tris-HCl buffer at pH 7.4. (F) Mass spectrum for binding of 2 eq **A06** with RB promoter G-quadruplex annealed in Tris-HCl buffer at pH 7.4.

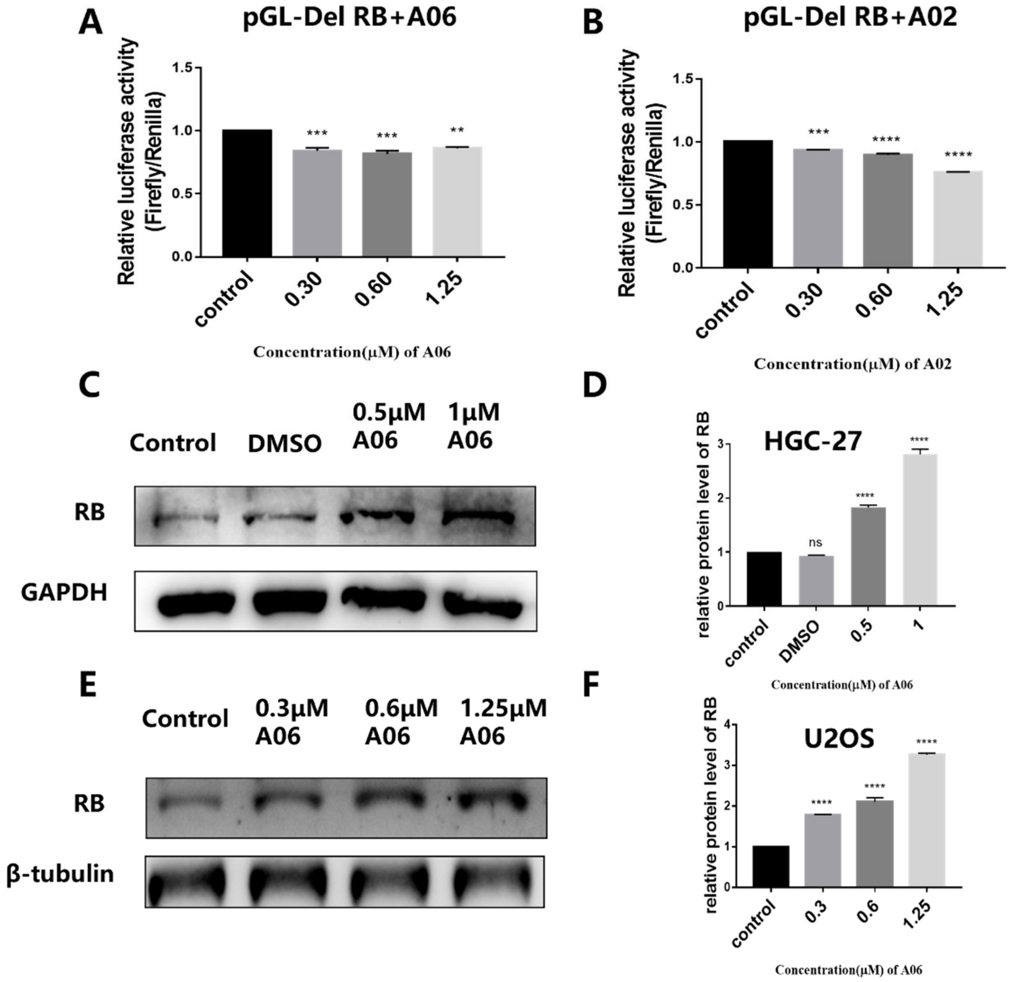

Figure S12. The effect of **A06** on RB gene transcription and translation. (A) Effect of **A06** on expression of pGL-Del RB plasmid luciferase. (B) Effect of **A02** on expression of pGL-Del RB plasmid luciferase. (C) WB bands of RB protein in HGC-27 cells incubated with different concentrations of **A06** for 24 h. (D) Histogram of gray value analysis of WB strip in Figure C. (E) WB bands of RB protein in U2OS cells incubated with different concentrations of **A06** for 24 h. (F) Histogram of gray value analysis of WB strip in Figure E. Experiments were repeated for three times, with data shown as mean ± SEM. ns means non-significant, ** means $P < 0.01$, *** means $P < 0.001$, and **** means $P < 0.0001$.

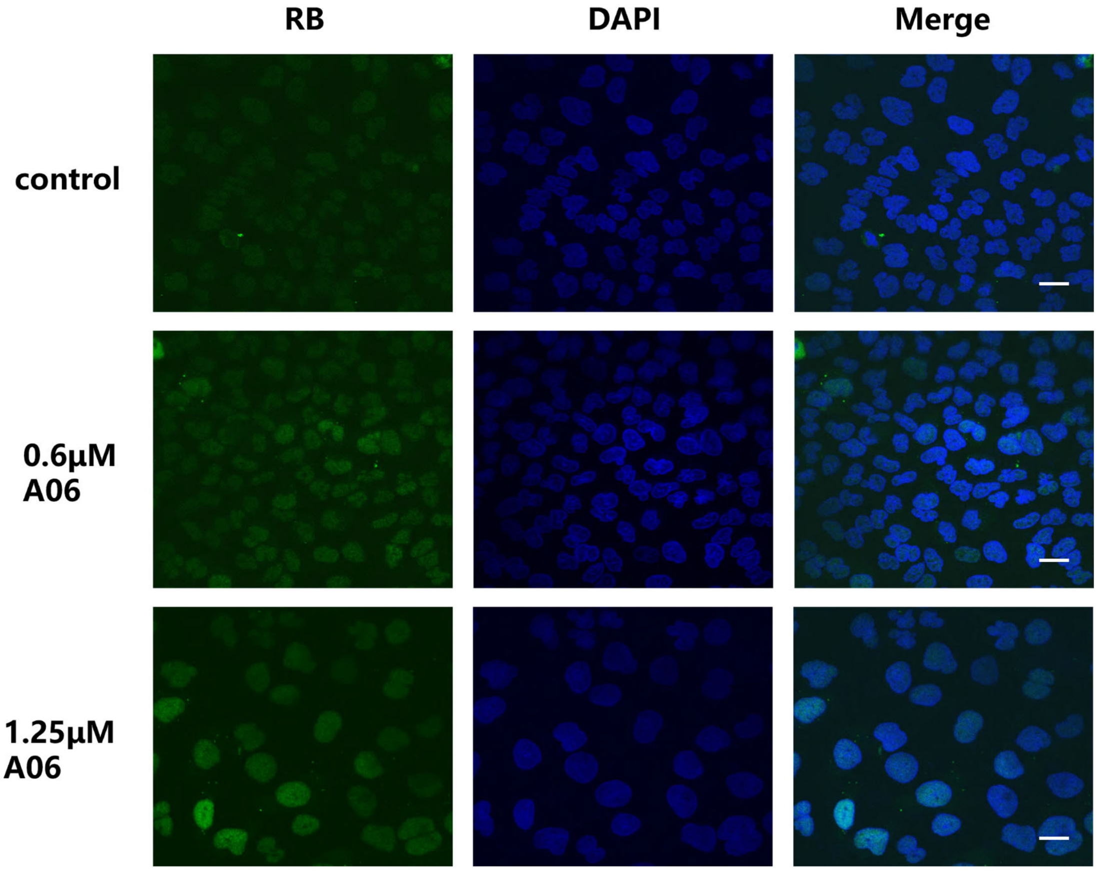

Figure S13. Immunofluorescence image of RB protein expressed in Hela cells incubated with different concentrations of **A06** for 48 h (Scale bars: 20 μm).

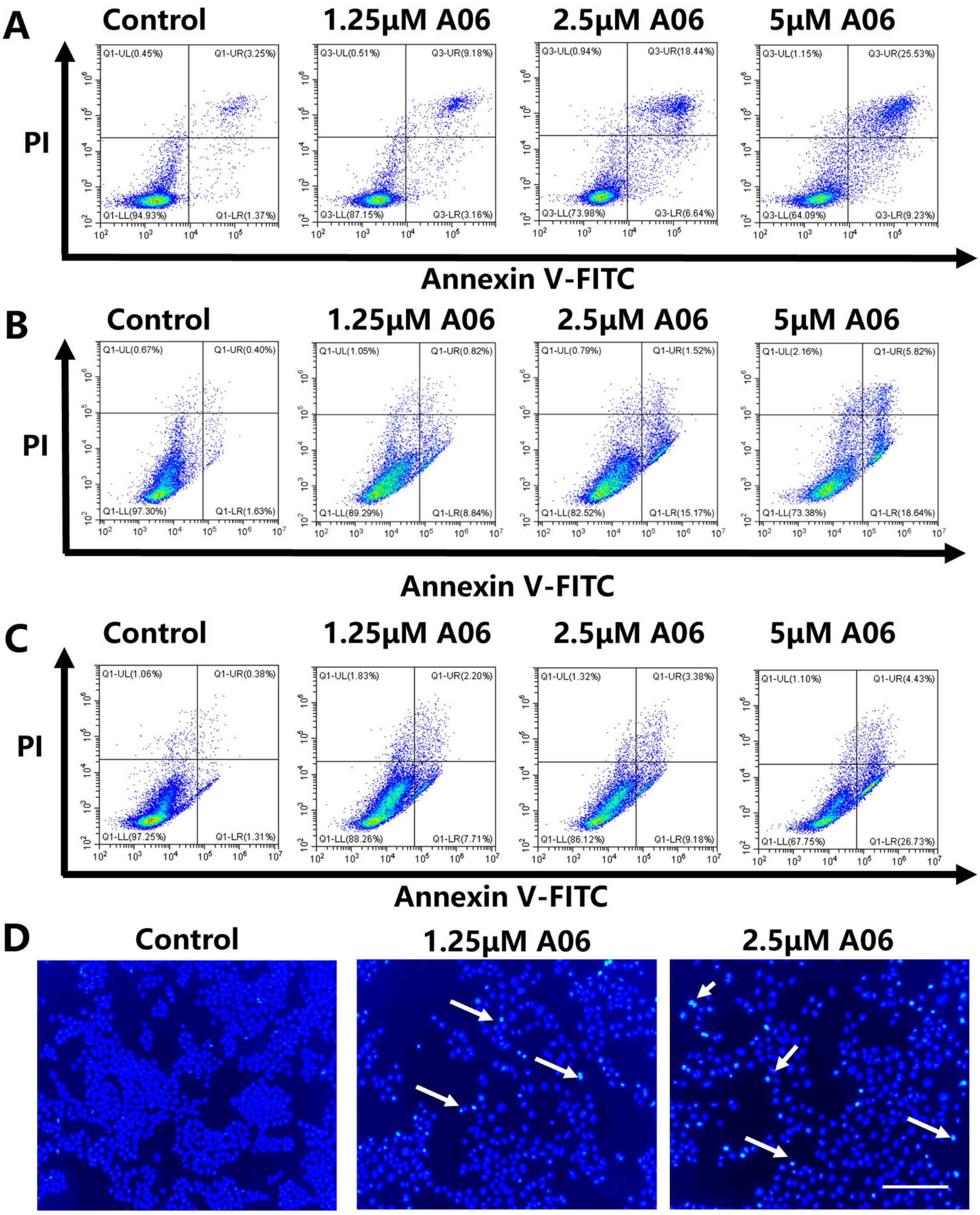

Figure S14. The anti-apoptotic effect of compound **A06** on different cancer cells. (A) Apoptosis results of Hela cells incubated with compound **A06** at increasing concentrations for 48h. (B) Apoptosis results of HGC-27 cells incubated with compound **A06** at increasing concentrations for 48h. (C) Apoptosis results of HCT116 cells incubated with compound **A06** at increasing concentrations for 48h. (D) Hoechst staining images of Hela cells incubated with compound **A06** at increasing concentrations for 48 h, with arrows indicating representative apoptotic cells (bright blue) (Scale bar: 200 μm).

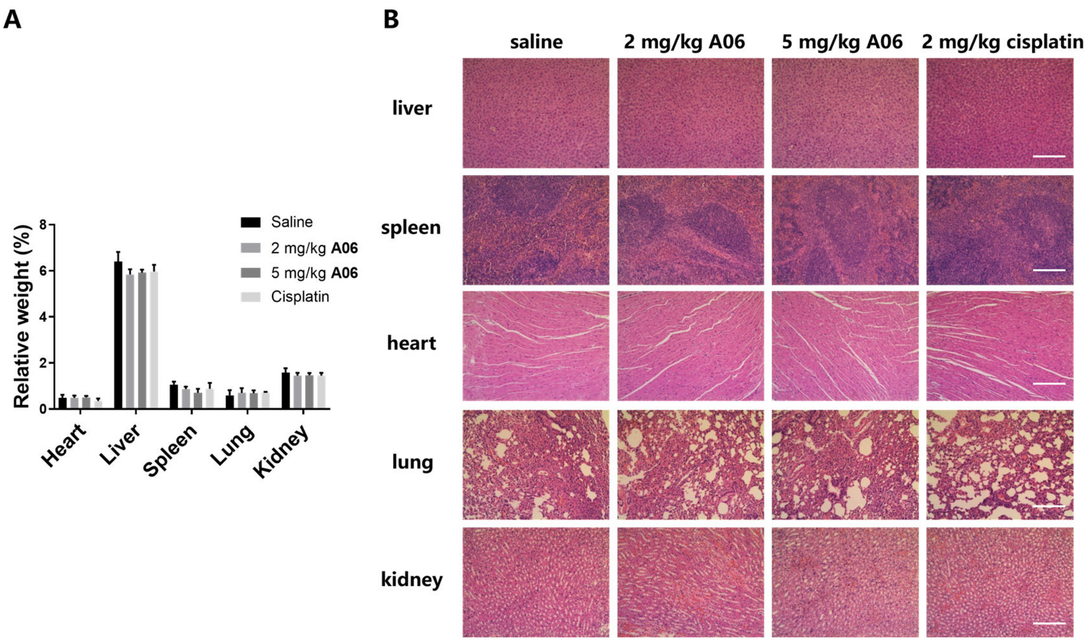

Figure S15. Comparison of vital organs for mice in different treatment groups. (A) The relative proportion of organs to body weight. (B) HE staining images of various organs (Scale bars: 200 μm).

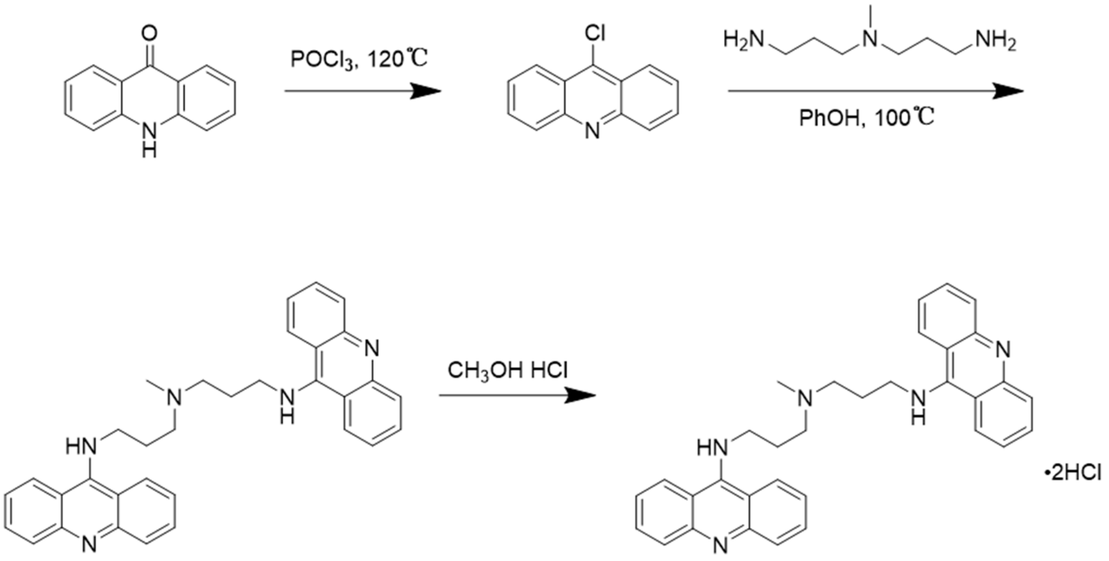

Scheme S1. Synthetic pathway for bisacridine derivative **A06** and **A06·HCl**. Reagents and conditions: (a) $POCl_3$, 100 °C, 3 h (yield 47%); (b) NH_2-L-NH_2, PhOH, 100 °C, 4 h (yield 91%); (c) MeOH, methanolic hydrochloric acid, r.t, 3 h (yield 93%).

Table S3. Oligonucleotides used in this study

Oligomer	Sequence
RB IM	5'- GCCGCCCAAAACCCCCCG -3'
RB G4	5'- CGGGGGGTTTTGGGCGGC -3'

ACC1 IM	5’-CCACCCGCCCGCCCGCCCGCCCAGC -3’
ACC1 G4	5’- GGGCGGGCGGGCGGGCGGG -3’
ACC2 IM	5’- TGACCCGCCCCCGCCCCGCCCCTGT -3’
ACC2 G4	5’- GGGGCGGGGCGGGGGCGGG -3’
braf IM	5’- CCCCCTTCCCCCGCTCCCCCCCGCACCCCC -3’
braf G4	5’- GGGGGTGCGGGGGGGAGCGGGGGAAGGGGG-3’
bcl-2 IM	5’- CAGCCCCGCTCCCGCCCCCTTCCTCCCGCGCCCGCCCCT 3’
bcl-2 G4	5’- GGGCGGGCGCGGGAGGAAGGGGGCGGG -3’
RET IM	5’- CCCCGCCCCGCCCCGCCCCTA- 3’
RET G4	5’- GGGGCGGGGCGGGGGGGGG-3’
c-jun IM	5’- TAACCCCCTCCCCCTCCCCCCTTTAAT -3’
c-jun G4	5’- AAGGGGGGAGGGGGAGGGGGTT -3’
kras IM	5’- CCTCCCCCTCTTCCCTCTTCCCACACCGCCCT -3’
kras G4	5’- AGGGCGGTGTGGGAAGAGGGAAGAGGGGGAGG -3’
Hif-1α IM	5’- GCCCGAGCGCGCCTCCGCCCTTGCCCGCCCCCTG -3’
Tel IM	5’- CCCTAACCCTAACCCTAACCCTAA -3’
VEGF G4	5’- GGGGCGGGCCGGGGGCGGGG -3’
c-myc IM	5’- CCTTCCCCACCCTCCCCACCCTCCCCA -3’
F-RB IM-T	5’-FAM- GCCGCCCAAAACCCCCCG -TAMRA-3’
F-RB G4-T	5’-FAM- CGGGGGGTTTTGGGCGGC -TAMRA -3’
5’-biotin RB IM	5’-biotin- GCCGCCCAAAACCCCCCG -3’
5’-biotin RB G4	5’-biotin- AGGGAGGGCGCTGGGAGGAGGG -3’
hairpin	5’- CGCGCGCGTTTTCGCGCGCG -3’
RB mut	5’- GTTGCTTAAAACCCCCCG -3’

Table S2. Equilibrium binding constants (K_D) determined by using SPR

Structure	compound	K_D (RB promoter i-motif)
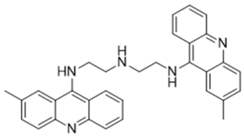 A02	**A02**	**9.24**
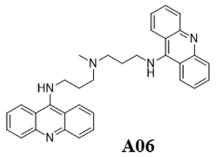 A06	**A06**	**2.44**
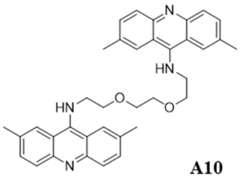 A10	**A10**	>50
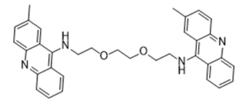 A11	**A11**	>50
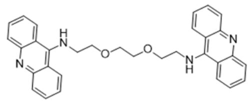 A12	**A12**	>50
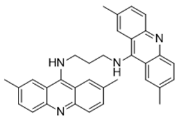 A13	**A13**	>50
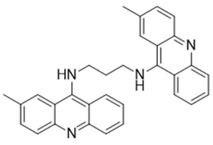 A14	**A14**	>50
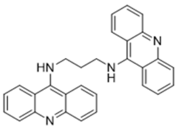 A15	**A15**	>50
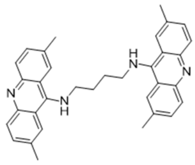 A16	**A16**	>50
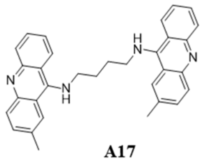 A17	**A17**	>50
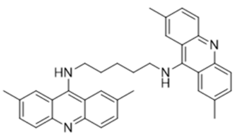 A18	**A18**	**15.9**
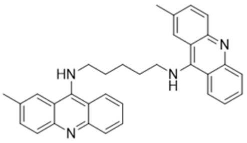 A19	**A19**	>50
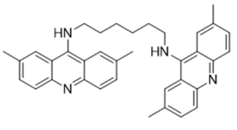 A20	**A20**	>50
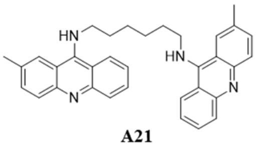 A21	**A21**	**43.2**
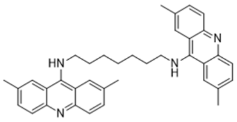 A22	**A22**	>50
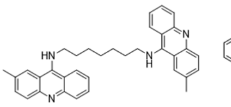 A23	**A23**	>50
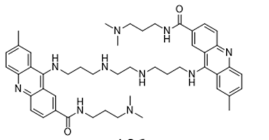 A26	**A26**	>50
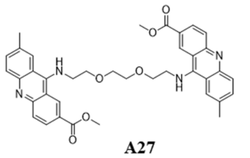 A27	**A27**	>50
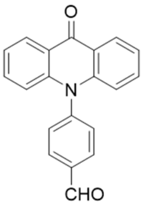 B1	**B1**	>50
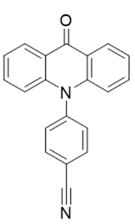 B2	**B2**	>50
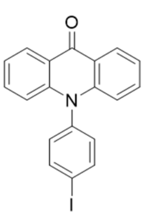 B3	**B3**	>50
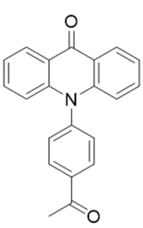 B4	**B4**	>50
	**B5**	>50
	**B6**	>50

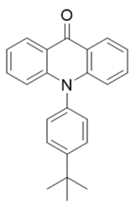 B5 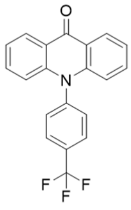 B6 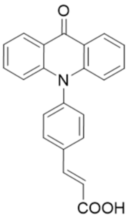 B7	**B7**	>50
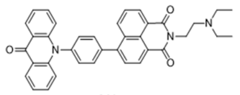 C69 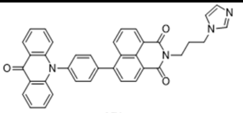 C70 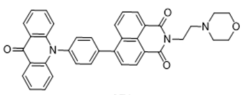 C71 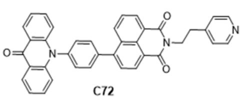 C72 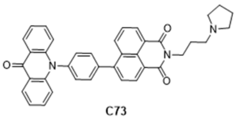 C73 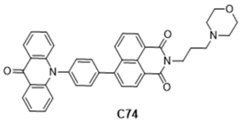 C74	**C69**	>50
	**C70**	>50
	**C71**	>50
	**C72**	>50
	**C73**	>50
	**C74**	>50
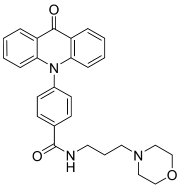 E40 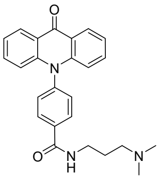 E41 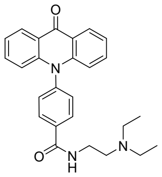 E42 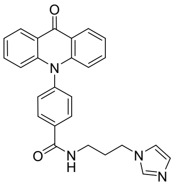 E43 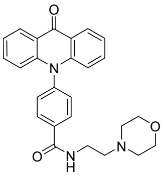 E44 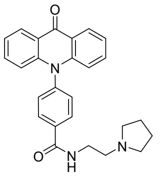 E45 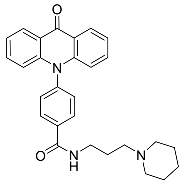 E46 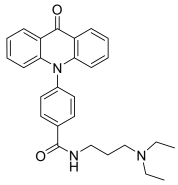 E47 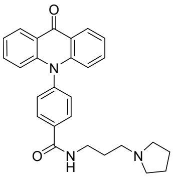 E48 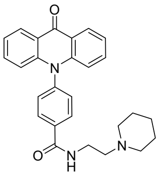 E49 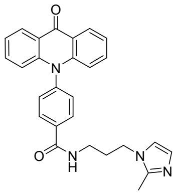 E50 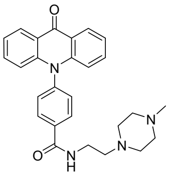 E51 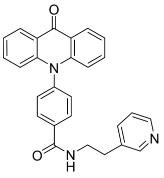 E52 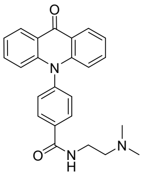 E53 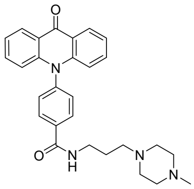 E54 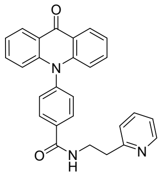 E55	**E40**	>50
	**E41**	>50
	**E42**	>50
	**E43**	>50
	**E44**	>50
	**E45**	>50
	**E46**	>50
	**E47**	>50
	**E48**	>50
	**E49**	>50
	**E50**	>50
	**E51**	>50
	**E52**	>50
	**E53**	>50
	**E54**	>50
	**E55**	>50
	**E56**	>50
	**E57**	>50
	**E58**	>50
	**E59**	>50
	**E60**	>50

<table>
<tr><td>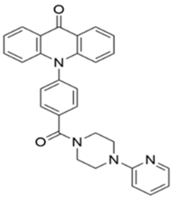 E56 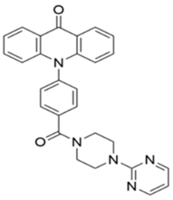 E57 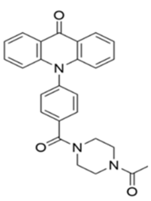 E58 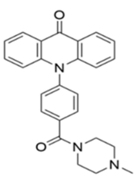 E59 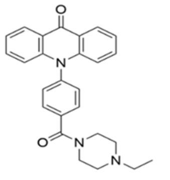 E60</td><td></td><td></td></tr>
<tr><td rowspan="15">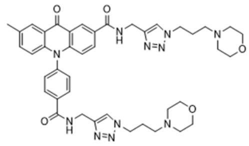 W06 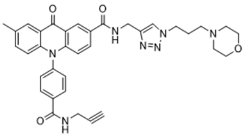 W06-1 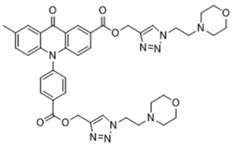 W07 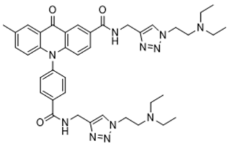 W10 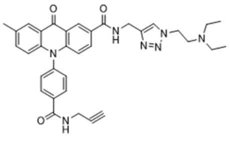 W10-1 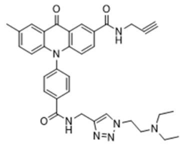 W10-2 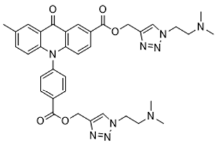 W11 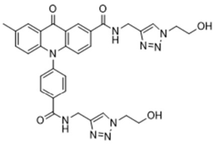 W12 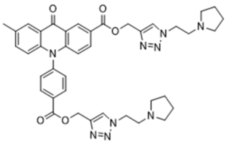 W13 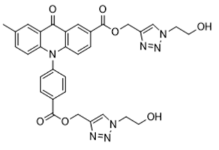 W14 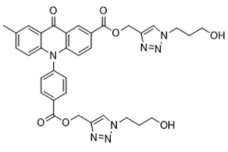 W15 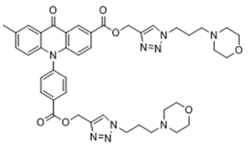 W16 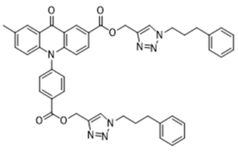 W17 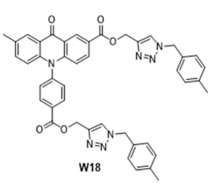 W18 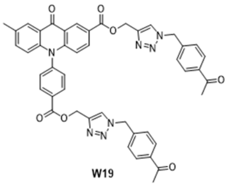 W19</td><td>W06</td><td>>50</td></tr>
<tr><td>W06-1</td><td>>50</td></tr>
<tr><td>W07</td><td>>50</td></tr>
<tr><td>W10</td><td>>50</td></tr>
<tr><td>W10-1</td><td>>50</td></tr>
<tr><td>W10-2</td><td>>50</td></tr>
<tr><td>W11</td><td>>50</td></tr>
<tr><td>W12</td><td>>50</td></tr>
<tr><td>W13</td><td>>50</td></tr>
<tr><td>W14</td><td>>50</td></tr>
<tr><td>W15</td><td>>50</td></tr>
<tr><td>W16</td><td>>50</td></tr>
<tr><td>W17</td><td>>50</td></tr>
<tr><td>W18</td><td>>50</td></tr>
<tr><td>W19</td><td>>50</td></tr>
<tr><td rowspan="7">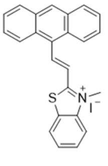 L1 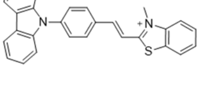 L2 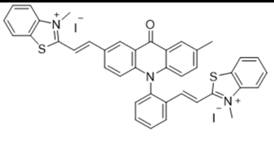 L3 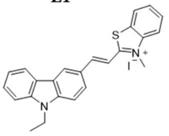 L4 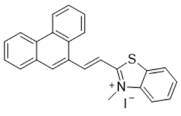 L5 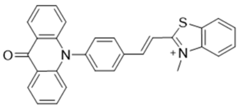 L6</td><td>L1</td><td>>50</td></tr>
<tr><td>L2</td><td>>50</td></tr>
<tr><td>L3</td><td>>50</td></tr>
<tr><td>L4</td><td>>50</td></tr>
<tr><td>L5</td><td>>50</td></tr>
<tr><td>L6</td><td>>50</td></tr>
<tr><td>L7</td><td>>50</td></tr>
</table>

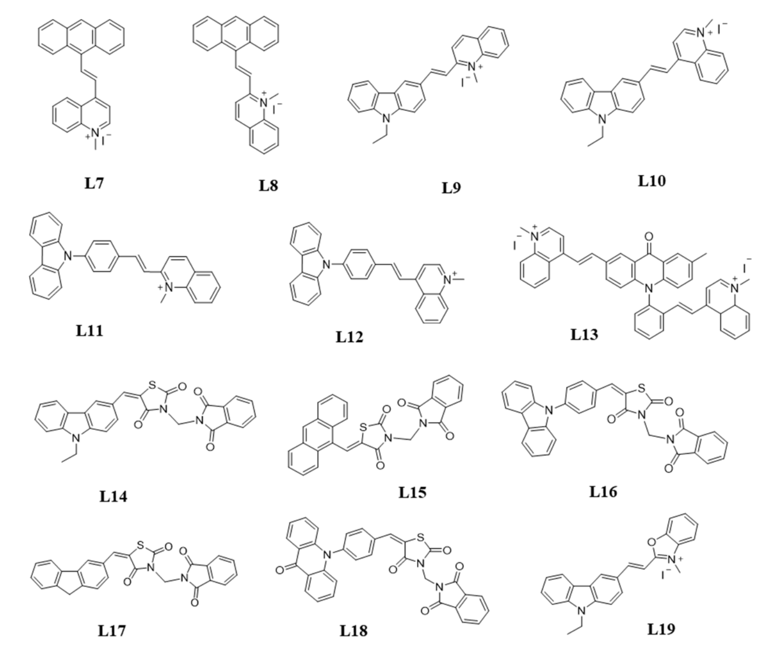	**L8**	**36.7**
	**L9**	>50
	**L10**	>50
	**L11**	>50
	**L12**	>50
	**L13**	>50
	**L14**	>50
	**L15**	>50
	**L16**	>50
	**L17**	>50
	**L18**	>50
	**L19**	>50

Table S3. The oligonucleotides for wild type or deleted RB promoter used in pGL-3 Basic plasmids, with underlined text indicating quadruplex forming sequence or mutant sequence.

Plasmids	Sequence
pGL-WT RB	GGTTTCCCAGTTTAATTCCTCATGACTTAGCGTCCCAGCCCGCGCAC CGACCAGCGCCCCAGTTCCCCACAGACGCCGGCGGGCCCGGGAGC CTCGCGGACGTGACGCCGCGGGCGGAAGTGACGTTTTCCCGCGGTT GGACGCGGCGCTCAGTTGCCGGGCGGGGGAGGGCGCGTCCGGTTT TTCTCAGGGGACGTTGAAATTATTTTTGTAACGGGAGTCGGGAGAGG ACGGGGCGTGCCCCGACGTGCGCGCGTCGTCCTCCCCGGCGCT CCTCCACAGCTCGCTGGCTCCCGCCGCGGAAAGGCGTCATGCCGCC CAAAACCCCCCGAAAAACGGCCGCCACCGCCGCCGCTGCCGCCGCG GAACCCCCGGCACCGCCGCCGCCGCCCCCTCCTGAGGAGGACCCA GAGCAGGACAGCGGCCCGGAGGACCTGCCTCTCGTCAGGTGAGCGA GCAGAGCCGCCGTCGCCTCACGCGGGAAGGGCGCCCCGGGTGT
pGL-Del RB	GGTTTCCCAGTTTAATTCCTCATGACTTAGCGTCCCAGCCCGCGCAC CGACCAGCGCCCCAGTTCCCCACAGACGCCGGCGGGCCCGGGAGC CTCGCGGACGTGACGCCGCGGGCGGAAGTGACGTTTTCCCGCGGTT GGACGCGGCGCTCAGTTGCCGGGCGGGGGAGGGCGCGTCCGGTTT TTCTCAGGGGACGTTGAAATTATTTTTGTAACGGGAGTCGGGAGAGG ACGGGGCGTGCCCCGACGTGCGCGCGTCGTCCTCCCCGGCGCT CCTCCACAGCTCGCTGGCTCCCGCCGCGGAAAGGCGTCATAAAAAC GGCCGCCACCGCCGCCGCTGCCGCCGCGGAACCCCCGGCACCGCC

	GCCGCCGCCCCCTCCTGAGGAGGACCCAGAGCAGGACAGCGGCCC<br>GGAGGACCTGCCTCTCGTCAGGTGAGCGAGCAGAGCCGCCGTCGCC<br>TCACGCGGGAAGGGCGCCCCGGGTGT
pGL-Mut RB	GGTTTCCCAGTTTAATTCCTCATGACTTAGCGTCCCAGCCCGCGCAC<br>CGACCAGCGCCCCAGTTCCCCACAGACGCCGGCGGGCCCGGGAGC<br>CTCGCGGACGTGACGCCGCGGGCGGAAGTGACGTTTTCCCGCGGTT<br>GGACGCGGCGCTCAGTTGCCGGGCGGGGGAGGGCGCGTCCGGTTT<br>TTCTCAGGGGACGTTGAAATTATTTTTGTAACGGGAGTCGGGAGAGG<br>ACGGGGCGTGCCCCGACGTGCGCGCGCGTCGTCCTCCCCGGCGCT<br>CCTCCACAGCTCGCTGGCTCCCGCCGCGGAAAGGCGTCATGTTGCT<br>TAAAACCCCCCGAAAAACGGCCGCCACCGCCGCCGCTGCCGCCGCG<br>GAACCCCCGGCACCGCCGCCGCCGCCCCCTCCTGAGGAGGACCCA<br>GAGCAGGACAGCGGCCCGGAGGACCTGCCTCTCGTCAGGTGAGCGA<br>GCAGAGCCGCCGTCGCCTCACGCGGGAAGGGCGCCCCGGGTGT

Table S4. Primers used for qRT-PCR

**Primer**	**Sequence**
GAPDH	forward：CCCTTCATTGACCTCAACTACATG
	reverse：TGGGATTTCCATTGATGACAAGC
RB	forward：ACCTTGAACCTGCTTGTCCTCT
	reverse：GGCTGAGGCTGCTTGTGTCT
VEGF	forward：GCTACTGCCATCCAATCGAG
	reverse：CTTGGTGAGGTTTGATCCGC
kras	forward：GTGGAGTATTTGATAGTGTTTAAC
	reverse：TGTATCAAAGAATGGTCCTGCA
Bcl-2	forward：TGTTGTTCAAACGGGATTCA
	reverse：GGCTGGGCACATTTACTGTT
braf	forward：TTCCGGAGGAGGTGTGGAAT
	reverse：TGCATCTAGCTTGCTGGTGT

Table S5. IC_{50} (μM) values were determined for effects of **A06** and **A02** on various tumor cells by using MTT assay

Compound	IC_{50} (μM)							
	Hela	SW480	Siha	A549	HCT116	HGC-27	HepG2	U2OS
**A06**	2.20	2.76	1.56	0.70	1.93	0.90	2.69	1.32
**A02**	16.01	17.59	10.96	11.72	10.11	nd	nd	nd

“nd” means not determined

^{1}H NMR and ^{13}C NMR spectra of bisacridine derivative **A06**

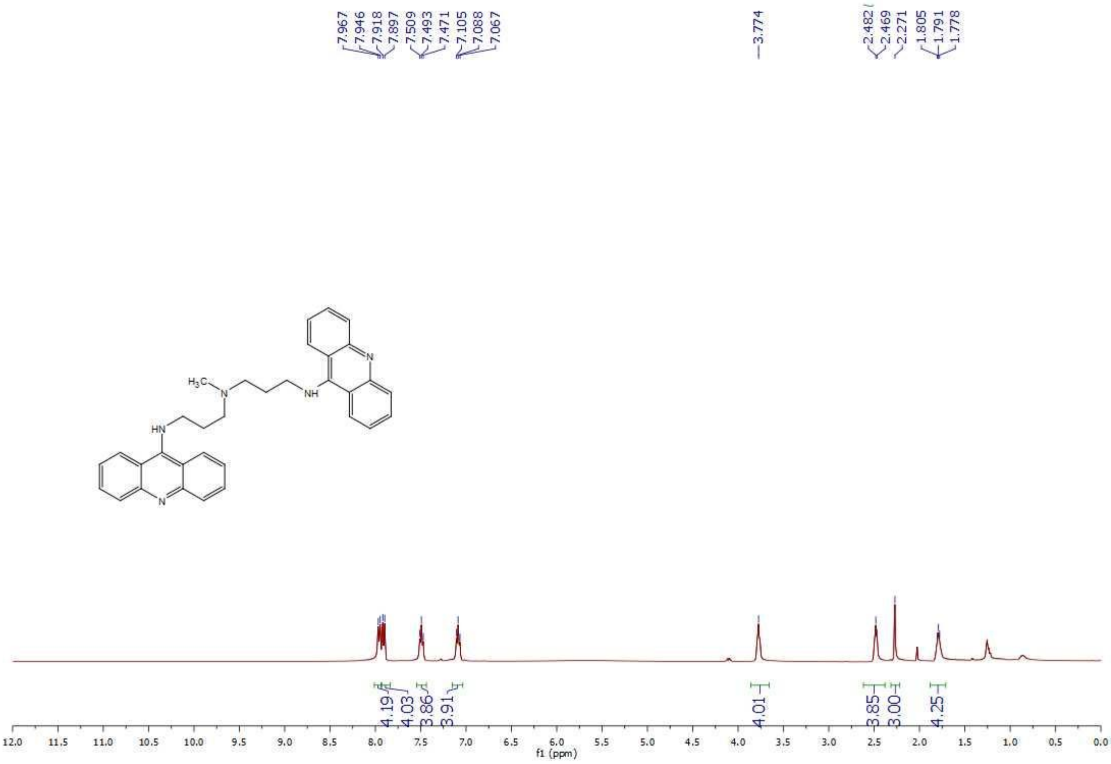

^{1}H spectrum of **A06**

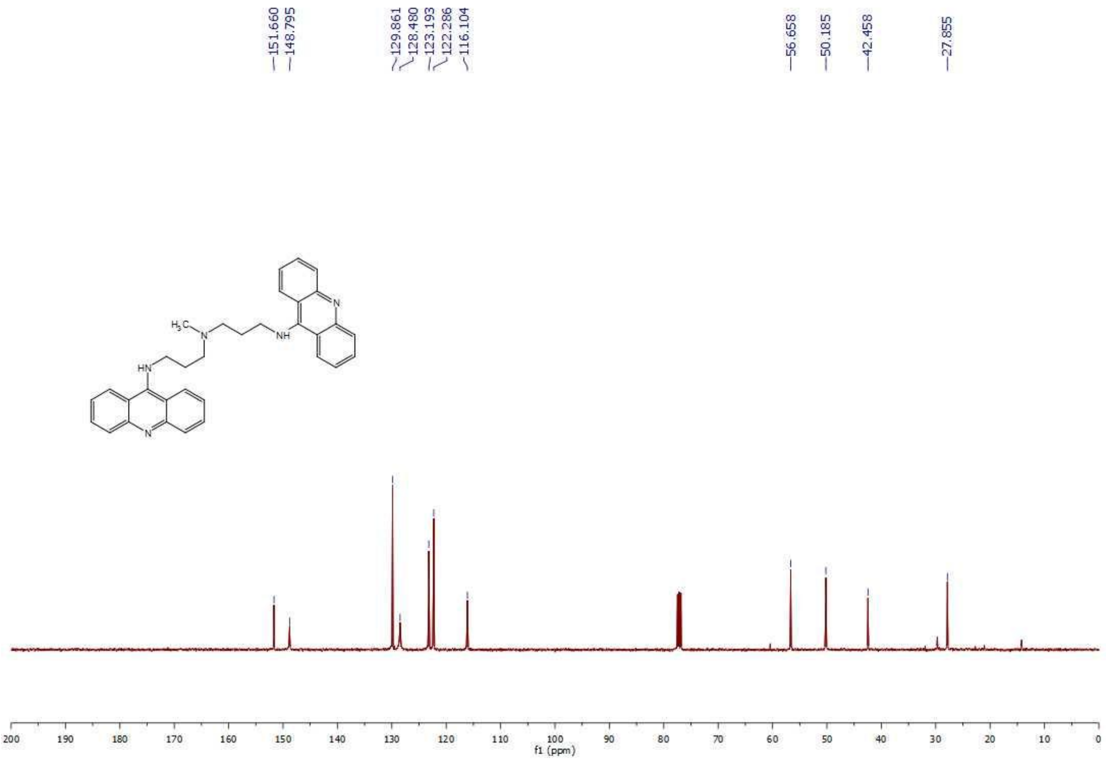

^{13}C spectrum of **A06**

^1H NMR and ^{13}C NMR spectra of bisacridine derivative **A02**

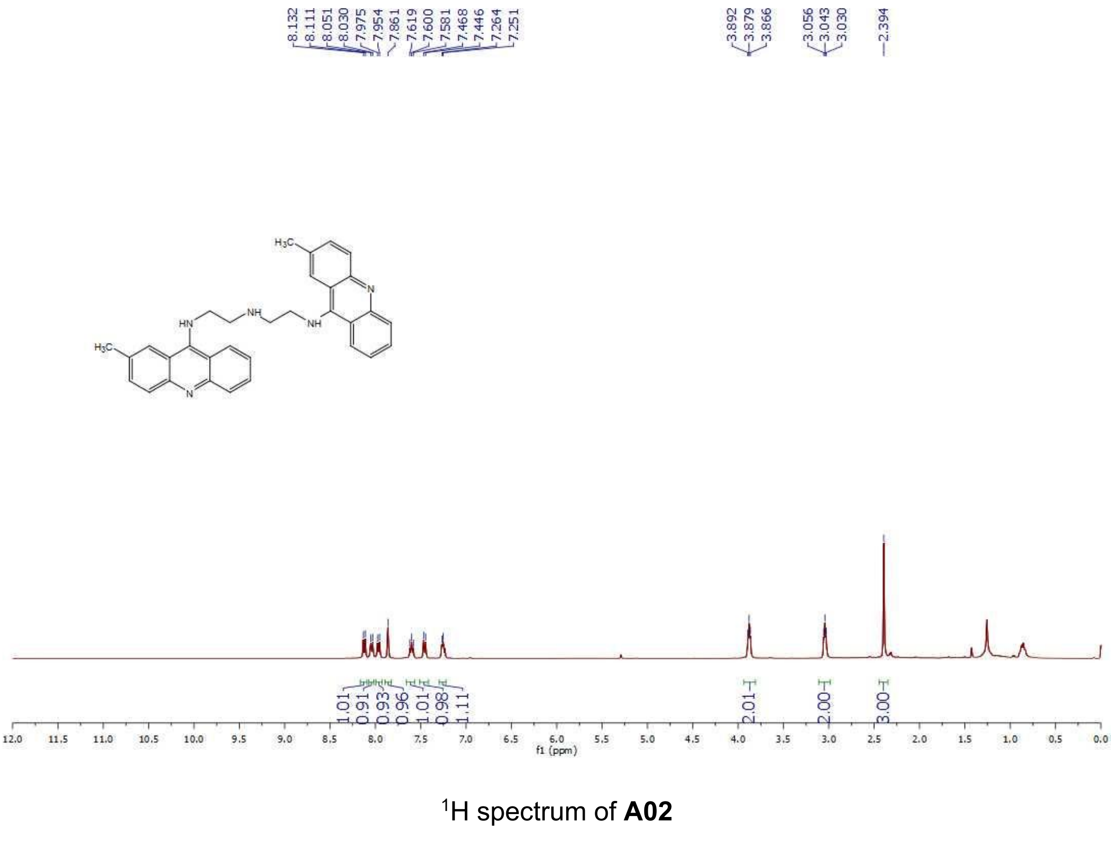

^1H spectrum of **A02**

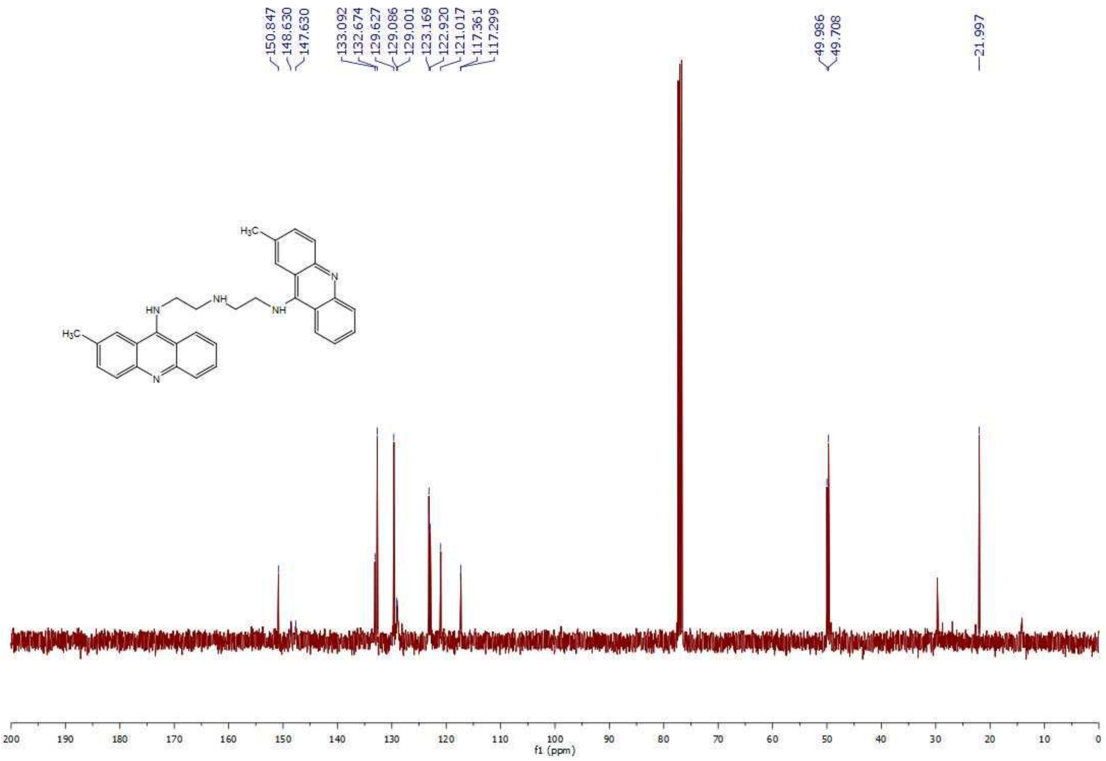

^{13}C spectrum of **A02**
